# A novel sequencing-based vaginal health assay combining self-sampling, HPV detection and genotyping, STI detection, and vaginal microbiome analysis

**DOI:** 10.1371/journal.pone.0215945

**Published:** 2019-05-01

**Authors:** Elisabeth M. Bik, Sara W. Bird, Juan P. Bustamante, Luis E. Leon, Pamela A. Nieto, Kwasi Addae, Víctor Alegría-Mera, Cristian Bravo, Denisse Bravo, Juan P. Cardenas, Glenn A. Carson, Adam Caughey, Paulo C. Covarrubias, José Pérez-Donoso, Graham Gass, Sarah L. Gupta, Kira Harman, Donna Marie B. Hongo, Juan C. Jiménez, Laurens Kraal, Felipe Melis-Arcos, Eduardo H. Morales, Amanda Morton, Camila F. Navas, Harold Nuñez, Eduardo Olivares, Nicolás Órdenes-Aenishanslins, Francisco J. Ossandon, Richard Phan, Raul Pino, Katia Soto-Liebe, Ignacio Varas, Patricia Vera-Wolf, Nathaniel A. Walton, Daniel E. Almonacid, Audrey D. Goddard, Juan A. Ugalde, Susan Zneimer, Jessica Richman, Zachary S. Apte

**Affiliations:** 1 uBiome, San Francisco, CA, United States of America; 2 Department of Biochemistry and Biophysics, University of California San Francisco, San Francisco, CA, United States of America; Istituto Nazionale Tumori IRCCS Fondazione Pascale, ITALY

## Abstract

The composition of the vaginal microbiome, including both the presence of pathogens involved in sexually transmitted infections (STI) as well as commensal microbiota, has been shown to have important associations for a woman’s reproductive and general health. Currently, healthcare providers cannot offer comprehensive vaginal microbiome screening, but are limited to the detection of individual pathogens, such as high-risk human papillomavirus (hrHPV), the predominant cause of cervical cancer. There is no single test on the market that combines HPV, STI, and microbiome screening. Here, we describe a novel inclusive vaginal health assay that combines self-sampling with sequencing-based HPV detection and genotyping, vaginal microbiome analysis, and STI-associated pathogen detection. The assay includes genotyping and detection of 14 hrHPV types, 5 low-risk HPV types (lrHPV), as well as the relative abundance of 31 bacterial taxa of clinical importance, including *Lactobacillus*, *Sneathia*, *Gardnerella*, and 3 pathogens involved in STI, with high sensitivity, specificity, and reproducibility. For each of these taxa, reference ranges were determined in a group of 50 self-reported healthy women. The HPV sequencing portion of the test was evaluated against the *digene* High-Risk HPV HC2 DNA test. For hrHPV genotyping, agreement was 95.3% with a kappa of 0.804 (601 samples); after removal of samples in which the *digene* hrHPV probe showed cross-reactivity with lrHPV types, the sensitivity and specificity of the hrHPV genotyping assay were 94.5% and 96.6%, respectively, with a kappa of 0.841. For lrHPV genotyping, agreement was 93.9% with a kappa of 0.788 (148 samples), while sensitivity and specificity were 100% and 92.9%, respectively. This novel assay could be used to complement conventional cervical cancer screening, because its self-sampling format can expand access among women who would otherwise not participate, and because of its additional information about the composition of the vaginal microbiome and the presence of pathogens.

## Introduction

A woman’s vaginal health is critical for her general well-being and reproductive success, and is in part determined by the vaginal microbiome composition, the presence of pathogens associated with sexually transmitted infections (STI), and the presence of human papillomavirus (HPV) types that can cause genital warts or cervical cancer. Current clinical vaginal health assays focus on the detection of STI or that of HPV, but there is no single test that combines these targets with vaginal microbiome analysis or with self-sampling.

The human vaginal microbiome has a unique composition compared to other microbial communities in the human body. In most healthy women, the vaginal microbiome is characterized by a low bacterial diversity and a dominance of lactobacilli, with low abundance of other bacterial genera [1,2]. Several vaginal microbial community types have been described, most of which are dominated by a single *Lactobacillus* species. Lactic acid produced by lactobacilli lowers the vaginal pH, which is believed to create an environment unfavorable for the growth of pathogenic bacteria [3]. Low numbers of vaginal lactobacilli have been associated with many health conditions, such as bacterial vaginosis [1,4–6], aerobic vaginitis [7], cervicitis [8], and STI [9–12]. The composition of a woman’s vaginal microbiome thus plays an important role in women’s health and reproductive success. Yet, the analysis of this microbial community is not part of regular health care for women. In the US, as in many other countries, most healthcare providers instead focus on the detection of high-risk human papillomavirus (hrHPV), the predominant cause for cervical cancer.

Cervical cancer is one of the major causes of cancer-related deaths in women, with an annual worldwide mortality of 250,000 [13,14]. hrHPV DNA can be detected in almost all (>99%) cervical cancer specimens, and HPV is therefore considered the predominant causative agent for cervical cancer [15,16]. Although HPV infection is the most common STI worldwide, not all HPV infections will lead to cancer. Firstly, certain HPV types have higher oncogenic risks than others. Of the over 170 different HPV genotypes known to date, twelve types have been classified as Group 1 human carcinogens; these include types 16, 18, 31, 33, 35, 39, 45, 51, 52, 56, 58, and 59 [17,18]. Together with other closely related HPV types, such as 66 and 68, which have been listed as probably or possibly carcinogenic, these are collectively called hrHPV types. hrHPV types 16 and 18 can be found in over 70% of cervical cancers [19] and the presence of these types is associated with the highest chance of developing cancer within 10 years [20]. However, other hrHPV genotypes have also been shown to cause cervical cancer, and especially among women of non-European descent [21]. In addition, many hrHPV infections are temporary and will be cleared within months of acquisition, without proceeding to pre-cancerous lesions [22]. Other HPV types, collectively called low-risk HPV (lrHPV), are not implicated in cervical cancer, but instead cause genital warts [23].

National cervical cancer screening programs are offered to women worldwide with a starting age between 20 and 35 years old [24]. Most of these programs involve an invitation for a Pap smear, in which a woman’s cervical cells are obtained by a physician for cytology [25], but additional molecular HPV testing is increasingly offered by health care providers as well [26]. In the United States, most healthcare providers follow the American College of Obstetricians and Gynecologists (ACOG) guidelines [27] or the U.S. Preventive Services Task Force (USPSTF) guidelines for women [28] to come in for a Pap smear, often with HPV testing, every three to five years, depending on age and risk factors. The most recent USPSTF guidelines, released in August 2018, even include HPV testing alone as a recommended option for women over 30 [29].

Several commercial kits have FDA pre-market approval for the molecular detection of HPV [30]. In a 2014 meta-analysis of 36 studies, the Qiagen *digene* Hybrid Capture 2 (HC2) assay was the most widely used [31]. In the HC2 assay, cervical specimens are denatured with sodium hydroxide, denatured viral DNA is hybridized with specific RNA probes, and RNA:DNA hybrids are subsequently detected with antibodies [32]. The HC2 test detects 13 hrHPV types, but does not report which specific type is present. Other HPV detection assays involve the amplification of viral DNA by Polymerase Chain Reaction (PCR). The most widely used primer pairs for HPV PCR detection are the GP5+/6+ primers [33] and the degenerate MY09/11 primers [34] or the PGMY09/11 primer pool [35], which are all based on conserved regions in the viral L1 open reading frame. The COBAS 4800 assay detects 14 hrHPV types using multiplex real-time PCR with specific probes; it reports the presence of HPV16, HPV18, or one of 12 remaining hrHPV types [36].

The vaginal microbiome is an emerging area of research in understanding the role of HPV infections and reducing the risk of cervical cancer [37]. Several studies suggest a relationship between the composition of the vaginal microbiota and the acquisition and persistence of HPV infection. For example, vaginal microbial diversity is increased during an HPV infection, with decreased levels of *Lactobacillus* species and an increased presence of other microbial members such as *Sneathia* species or *Gardnerella vaginalis* [38–42]. In addition, certain microbiota compositions are associated with increased clearance of detectable HPV [40].

In this study, we tested the feasibility of a novel assay, that combines the detection and identification of HPV DNA, STI-associated pathogens, and microbiome analysis on samples obtained through self-sampling. We validated the performance of marker gene amplification and sequencing to detect the presence and relative abundance of 31 clinically important bacterial targets with high precision and accuracy. In addition to detecting *Lactobacillus*, *Sneathia*, and *Gardnerella* spp., this test detects STI-associated pathogens including *Chlamydia trachomatis*, *Mycoplasma genitalium*, and *Neisseria gonorrhoeae*, which combined infect over 150 million people each year and cause genital tract infections and cervicitis [10–12]. The performance of a novel amplification and sequencing-based strategy for HPV detection and type-specific identification was compared to that of the most widely used test for HPV detection in cervicovaginal specimens, the *digene* HC2 test. This assay was not developed to be employed in a clinical setting, but intended to complement, rather than replace, current healthcare guidelines for in-clinic cervical cancer screening.

## Materials and methods

### Study participants and sample collection

This study was approved under a Human Subjects Protocol provided by an independent IRB (E&I Review Services, IRB Study #13044, 05/10/2013). E&I is fully accredited by the Association for the Accreditation of Human Research Protection Programs, Inc. (AAHRPP), with Registration # IRB 00007807. uBiome undergoes a yearly voluntary continuing review by E&I Review Services to determine that the project meets the same scrutiny of human subjects protections as projects conducted at research institutions. The IRB board membership of E&I Review Services is consistent with the Code of Federal Regulations (CFR) requirements of 21 CFR 56.107 and 45 CFR 46.107. Where required, specimens used in this study consisted of vaginal samples from women who had signed an informed consent to have their samples used for research. All participants were 18 years or older.

A vaginal self-collection kit was sent to each participant’s home address, consisting of a sterile swab, a tube with sterile water, a tube with zirconia beads in a proprietary lysis and stabilization buffer that preserves the DNA for transport at ambient temperatures, and sampling instructions (S1 Fig, included in the Supplementary Materials). Participants were instructed to wet the swab with the sterile water, insert the swab into the vagina as far as is comfortable, make circular movements around the swab’s axis for 1 minute (min), and then stir the swab for 1 min into the tube with lysis buffer and beads. After shaking the tube for 1 min to homogenize, the tube was then shipped by the participants to the laboratory by regular mail.

For the determination of the reference ranges of the 31 bacterial targets, a set of 50 vaginal specimens, each from a different woman (average age 48.4 ± 15.6 years; range 23 to 79 years), was selected. Inclusion criteria were the following: completion of the voluntary health survey that every woman was invited to participate in, and no self-report of any of the following conditions: bacterial vaginosis, cervical cancer, genital herpes or warts, urinary tract infection, or infection with HPV, *C*. *trachomatis*, *T*. *pallidum*, or yeast. In addition, all of these women reported no antibiotic usage in the six months before sampling.

A different set of specimens from 87 women was used to compare the performance of sampling with the *digene* collection device (Qiagen, Gaithersburg, MD, USA) and DNA extracted from samples collected with swabs. For this subset, women were asked to self-sample 2 vaginal specimens within 15 minutes. The first specimen was collected by using the *digene* collection device, which consists of a cervical brush and a *digene* transport tube with Specimen Transport Medium (STM). The second specimen was collected using a pre-wetted swab and resuspended in a collection tube with lysis buffer and beads, as described above, and used for DNA extraction.

For use in spiking and intra-run technical repeatability experiments described below, homogenized “vaginal pools” were created by combining 96 vaginal samples derived from 11 or 16 individuals who sampled themselves multiple times. These pools were created to make a relative large amount of a homogenized complex matrix similar to real biological samples. The pool created from samples taken by 16 subjects was used to test the synthetic DNA in spiking experiments, while the pool of samples taken by 11 subjects was used in the technical repeatability experiment (see below).

An additional set of 718 vaginal specimens were selected to compare the performance of the *digene* HC2 HPV test using the hrHPV probe (601 specimens) and lrHPV probe (148 specimens; overlap of 31 specimens), respectively, versus that of the amplification and sequence-based HPV type identification described in this study. Of these, 361 samples were from subjects who consented, while the remaining samples were residual clinical samples that were analyzed anonymously.

### Positive STI control samples

Ten de-identified cervicovaginal swab specimens of known STI pathogen status were obtained through a commercial source (iSpecimen, Lexington, MA). Five of these samples were reported to be positive for *C*. *trachomatis* and negative for *N*. *gonorrhoeae*, while a second set of five samples were negative for *C*. *trachomatis* and positive for *N*. *gonorrhoeae*. Each sample was tested in five replicates for DNA extraction, 16S rRNA gene amplification, and target identification as described below. To confirm the presence of *M*. *genitalium* in 13 samples that tested positive and 9 samples that tested negative for this pathogen in the assay using the 16S rRNA gene amplification described in this study, a PCR was performed using primers on the MgPa adhesin gene [43].

### In silico 16S rRNA gene target performance metrics

**Fig 1:**
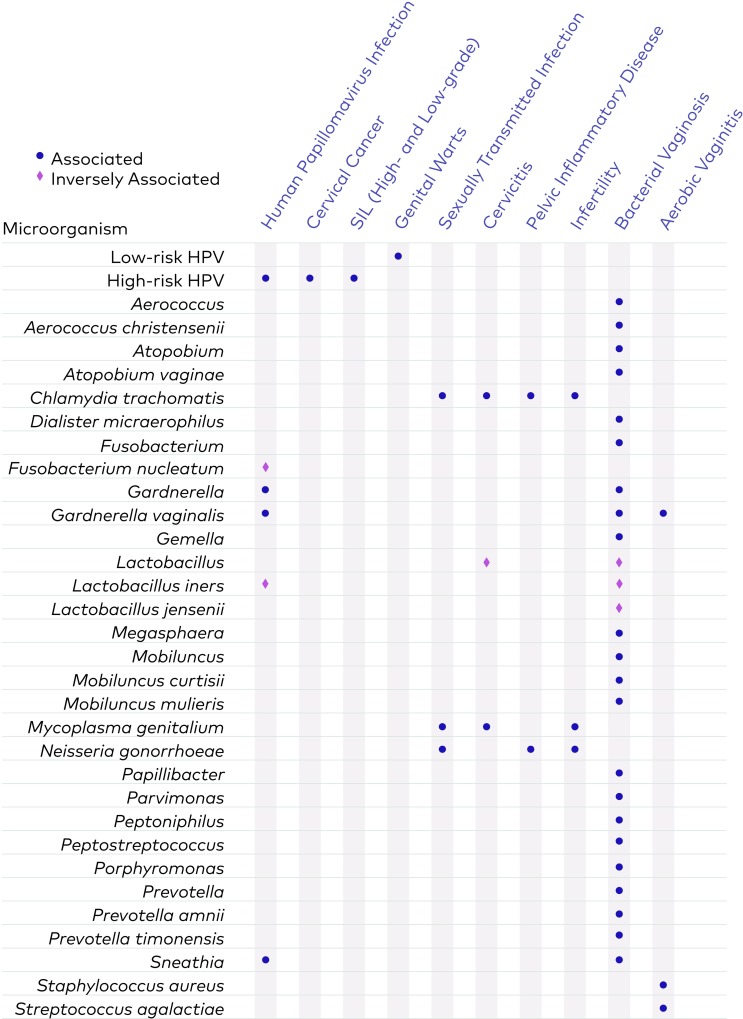
The 31 bacterial targets and HPV targets covered by the assay and their associated health conditions. Dark blue dots indicate targets positively associated with the conditions, while pink diamonds indicate inverse associations. See S1 Table in the Supplementary Materials for more detailed information about e.g. the HPV genotypes included and a list of references.

The assay includes 31 bacterial targets with clinical relevance for women’s reproductive tract health, which were identified through an exhaustive literature search (Fig 1). The most relevant associations between health conditions and the vaginal microbiota were narrowed down by choosing associations with high statistical significance that were found in humans subjects, not in laboratory animals or bioreactors, but performed on case/control, cohorts or randomized studied population. These include bacterial vaginosis [1,4–6], aerobic vaginitis [7], pelvic inflammatory disease [44], and sexually transmitted infections [10–12]. A complete list of these associations and references are provided in S1 Table. For each bacterial taxon intended to be included in this assay, using a process similar to that described in Almonacid et al. [45], we determined *in silico* performance metrics for identification of each taxa (sensitivity, specificity, positive and negative predictive value). Briefly, sequences assigned to each taxon in the SILVA database (Version 123) [46] were considered to be real positives for that taxon. Then, assuming amplification with up to two mismatches with the primers used, we identified for each taxa the sequences that would produce an amplicon, and evaluated whether that amplicon is unique to the taxon of interest (ti) or also shared by sequences from different taxa (dt). The number of true positives (TP), true negatives (TN), false positives (FP) and false negatives (FN) was computed for different tolerance ratios for the quotient dt/ti, and subsequently *in silico* performance metrics were assessed. Of the 72 bacterial targets initially selected, the 31 targets selected for the assay had all four *in silico* performance metrics above 90% (S2 Fig; S2 Table).

### In silico HPV target performance metrics

In addition to the 31 bacterial targets, hrHPV and lrHPV targets were selected for inclusion in the assay, based on their published association with cervical cancer lesions or genital warts (Fig 1, S1 Table). HPV reference genomes were downloaded in August 2017 from the PaVE database, which is a repository of curated and annotated HPV genomes [47,48]. Only revised and recognized sequences (180 HPV genomes) were used for an *in silico* PCR amplification using a set of 15 forward and 6 reverse primers (described below) targeting the L1 gene and allowing up to 4 mismatches between primers and target sequences. Under these conditions, the L1 genes from 118 HPV genomes could be amplified *in silico*. Of these, 19 HPV genomes, including 14 hrHPV types (16, 18, 31, 33, 35, 39, 45, 51, 52, 56, 58, 59, 66, 68) and 5 lrHPV types (6, 11, 42, 43, 44) were selected based on their association with health conditions according to literature (S1 Table). In order to evaluate the performance metrics for identification of the HPV targets, sequences of the L1 segment of HPV genomes from the NCBI database were used. The search was filtered to sequences with length in the range 1,500–10,000 bp and with correct assignment of the type of the HPV (4177 sequences). These sequences were amplified *in silico* using the primers described below. Following these steps, we generated 161,398 amplicons. These sequences were mapped using VSEARCH [49] at 95% of identity against an HPV amplicon reference database consisting of the amplicons produced by the reference genomes in PaVE for the 19 HPV types included in our assay. The performance metrics were calculated as described above for the 16S rRNA gene targets. Briefly, the correct assignment of an *in silico* NCBI amplicon against the reference was counted as a true positive and an incorrect assignment was considered as a false negative. Also, we considered as a false negative any genome from NCBI that our primers could not amplify. According to this, the 19 HPV types obtained values for sensitivity, specificity, positive predictive value (PPV) and negative predictive values (NPV) above 90% (S3 Table).

### In vitro validation of bacterial targets

To test our ability to identify members of each of the 31 bacterial targets, synthetic DNAs (sDNAs or gBlocks, Integrated DNA Technologies, Inc., Coralville, IA), were designed encompassing the V4 region of the 16S rRNA gene including primer regions, based on a SILVA representative sequence, plus 75 additional bases to both the 5’ and 3’ side, with one sDNA per target (S4 Table). The SILVA representative sequence per taxa was chosen by performing an all-against-all sequence comparison of all sequences in a taxa, and identifying as representative the sequence that shared the highest similarity with the largest number of sequences in the set.

To validate that each target could be detected in a vaginal swab specimen, 3 ng of each sDNA was spiked into 500 μl aliquots of a vaginal pool, created by combining 96 vaginal specimens from 16 women included in this study, and DNA was extracted from each spiked vaginal pool (see below). Each spike-in experiment was performed in triplicate. Subsequently, bacterial targets were detected by amplification using PCR targeting the 16S rRNA gene, sequencing, and a bioinformatics pipeline described below. Each target included in the final panel was detected above limit of detection (LOD) (see below) in each of the triplicate spiked-in amplification reactions performed on the extracted DNA from the vaginal pool (not shown).

In addition, the LOD of each target in a complex background of other targets was determined according to published guidelines [50]. First, to check for potential contamination, we calculated a limit of blank (LOB), which was calculated using a set of 77 blank wells of a 96-well PCR plate where wells of the first row and first column of the plate each contained 200 pg/μl of synthetic 16S rRNA gene DNA from different targets. The LOB was set as the average number of reads in these blank wells (18.57 reads) plus 1.65 standard deviations (29.70 reads), thus at 48.27 reads. To calculate the LOD of the bacterial target, pools of bacterial sDNAs were mixed in different ratios. To create these mixes, each bacterial sDNA was randomly assigned to one of two pools, A and B, that each contained sDNAs in equimolar amount. Each pool was serially diluted in PCR grade water. Pool A dilutions were mixed 1:1 with undiluted Pool B and vice versa. All pool A/B combinations were used in triplicate for DNA extraction, amplification, and sequencing as described below. For each target, the LOD was defined as the lowest concentration of sDNA where at least two of the three replicates contained at least 2 reads for that target in a sample with 10,000 reads or more. Using this LOD, we calculated a lower threshold for detection for each taxa at its LOD as the LOB (48.27) plus the standard deviation of the taxa at LOD * 1.65. This threshold is used to correctly assign a taxa as identified in a sample at or above its LOD.

For targets that had both a species and a genus level sDNA present in the mixed pools A and B, a bioinformatic correction was applied. The total reads for a genus-level target for which a species within that genus was also present in the mixed pools, was defined as the total measured reads for the genus and subtracting all those reads corresponding to species-level targets belonging to that genus in the same pool mix, i.e., only reads that match to a genus and not to a species level were finally assigned to the genus.

### In vitro validation of HPV targets

To test the ability of our assay to detect and genotype HPV targets, fragments of the L1 gene of approximately 600 bp long were ordered for each of the representative sequences of 19 HPV types in the PaVE database as sDNAs (gBlocks, Integrated DNA Technologies, Inc.). To represent hrHPV type 68, two sDNAs were ordered, 68a and 68b. The sequences of the 20 gBlocks representing 19 HPV types (14 hrHPV and 5 lrHPV) are listed in S5 Table. To validate that each target could be detected in a vaginal swab specimen, 3 ng of each HPV sDNA was spiked into 500 μl aliquots of a vaginal pool created by combining 96 vaginal specimens from 16 women included in this study, and DNA was extracted from each spiked vaginal pool (see below). Subsequently, the spiked HPV targets were detected by amplification using the PCR targeting the L1 gene and bioinformatics pipeline described below. Each spike-in experiment was performed in triplicate. Each HPV target was detected above the LOD (see below) in each of the triplicate spiked-in amplification reactions performed on the extracted DNA from the vaginal pool (not shown). Each target had a ratio > 0.1 for the number of HPV-assigned reads divided by the total number of normalized reads assigned to an internal spike-in control (see below).

To determine the LOD of HPV targets, 10-fold serial dilutions of the sDNAs representing HPV targets were made in nuclease-free water, ranging from 10^5^ to 10^2^ molecules per μl. Dilutions of one target were inversely combined with dilutions of another target, forming different pairs of HPV sDNAs. Each dilution pair was used directly as template for PCR in triplicate as described below.

### DNA extraction and amplification targeting 16S rRNA and HPV L1 genes

DNA was extracted from vaginal specimens, pools thereof, or sDNA dilutions in tubes containing lysis/stabilization buffer as described previously [45]. For 16S rRNA gene amplification, extracted DNA was used as the input of a one-step PCR protocol to amplify the V4 variable region of the 16S rRNA gene. This PCR contained universal primers 515F and 806R [45,51], both with sample-specific indices and Illumina tags. PCR was performed as described before [45]. Following amplification, DNA was pooled by taking the same volume from each reaction.

For HPV amplification, extracted DNA was used as the input of a PCR protocol to amplify the HPV L1 gene. To each sample, sDNA with a randomized HPV type 16 sequence was added as an internal positive control. The first PCR mix contained a pool of previously described HPV specific primers [35,52], and two new primers, HPV_RSMY09-LvJJ_Forward: 5’ CGTCCTAAAGGGAATTGATC, and HPV_PGMY11-CvJJ_Reverse: 5’ CACAAGGCCATAATAATGG. All these primers contained sequencing adaptor regions. The PCR products from the first amplification round were used as input for a second PCR containing sample-specific forward and reverse indices and Illumina tags. PCR products from this second step were pooled for sequencing.

The 16S rRNA gene and HPV PCR consolidated library pools were separately quantified by qPCR using the KAPA Library Quant Kit (Bio-Rad iCycler qPCR Mix) following the manufacturer’s instructions using a BioRad MyiQ iCycler. Sequencing was performed in a paired-end modality on the Illumina NextSeq 500 platform rendering 2 x 150 bp paired-end sequences.

### Sequence analysis and taxonomic annotation for bacterial targets

After sequencing, demultiplexing of reads according to sample-specific barcodes was performed using Illumina’s BCL2FASTQ algorithm. Reads were filtered using an average Q-score > 30. Forward and reverse 16S rRNA gene reads were appended together after removal of primers and any leading bases, and clustered using version 2.1.5 of the Swarm algorithm [53] using a distance of one nucleotide and the “fastidious” and “usearch-abundance” flags. The most abundant sequence per cluster was considered the real biological sequence and was assigned the count of all reads in the cluster. The representative reads from all clusters were subjected to chimera removal using the VSEARCH algorithm [49]. Reads passing all above filters (filtered reads) were aligned using 100% identity over 100% of the length against the true positive 16S rRNA gene sequences identified *in silico* from SILVA for each of the 31 taxonomic groups targeted by the assay as described above (S4 Table). The relative abundance of each taxon was determined by dividing the count linked to that taxa by the total number of filtered reads.

### Sequence analysis and taxonomic annotation for HPV targets

Raw sequencing reads were demultiplexed using BCL2FASTQ. Primers were removed using cutadapt [54]. Trimmomatic [55] was used to remove reads with a length less than 125 bp, and a mean quality score below 30. After that, forward and reverse paired reads were joined using custom in-house scripts and converted to a fasta file. Identical sequences were merged and written to a file in fasta format and sorted by decreasing abundance using—derep_fulllength option in VSEARCH [49]. Target sequences in the fasta files were compared to the fasta-formatted query database sequences (19 HPV target sequences) using the global pairwise alignment option with VSEARCH, using 95 percent sequence identity, to obtain the counts for each HPV type within a different sample.

The HPV portion of the assay was considered positive if the number of sequence reads assigned to the specific HPV types was above the threshold at the limit of detection, and greater than a previously defined cutoff. To set this cutoff, two normalization steps were employed. First, according to *in silico* PCR amplification, a different number of combinations of primers amplify different HPV targets (e.g. HPV16 is amplified using 66 different combinations, while HPV43 is amplified with just 10 combinations), reflecting the sequence variability within the primer binding site among HPVs. This also means that the spiked-in internal control and the target HPV have different amplification efficiencies. To avoid this bias, the internal control (which has the primer sites for HPV16) is normalized for the amplification factor (number of primer combinations that generate an amplicon) of each HPV type. The number of HPV-assigned reads was divided by the total number of normalized reads assigned to the spike, and a sample was considered HPV-positive if that ratio was above 0.1, which corresponds to approximately 500 target molecules.

### Intra- and inter-run precision

Intra-run technical repeatability was assessed by including nine replicates of the same vaginal pool (consisting of 96 vaginal samples derived from 11 individuals) into the same DNA extraction, 16S rRNA gene amplification, and sequencing run. This experiment was then repeated in two additional sequencing runs to yield three sets of nine replicate samples analyzed within the same run. In addition, inter-run technical reproducibility was performed by processing three replicates of a set of 18 vaginal samples on three different days by three different operators. Samples included in the analysis were those that had at least 10,000 reads and where at least two of the three replicates were present (11 sets).

Comparison of the results, both intra- and inter-run, were done using the raw counts of the 31 bacterial species- and genus-level targets. Data was processed using the R-package Phyloseq [56], and visualized using Principal Coordinates Analysis (PCoA), based on a distance matrix calculated using the Bray-Curtis method.

### *digene* HC2 hrHPV test on *digene* tubes or on extracted DNA

The *digene* HC2 HPV detection assay (Qiagen) was used as a reference to validate the hr- and lrHPV portions of the assay. The High-Risk HPV Probe in the *digene* HC2 HPV test detects hrHPV types 16, 18, 31, 33, 35, 39, 45, 51, 52, 56, 58, 59, and 68, while the Low-Risk HPV Probe in the *digene* test detects lrHPV types 6, 11, 42, 43, and 44. The vaginal health test described here detects all these types plus hrHPV type 66. The *digene* HC2 assay is intended to be used directly on cervical samples collected in the *digene* STM transport tube. In order to validate the use of the *digene* kit on extracted vaginal DNA, we first compared the performance of the *digene* HC2 assay on a set of 87 self-sampled, paired vaginal samples, i.e. a *digene* brush resuspended in an STM transport tube, as well as DNA extracted from a vaginal swab resuspended in lysis and stabilization buffer according to the manufacturer’s instructions. Negative and positive controls and calibrators included in the kit were processed within each 96-well assay, and used for assay validation and cutoff, as per instructions. A specimen was considered positive if its chemiluminescence measurement (Relative Light Units, RLU) was higher than or equal to that of the assay’s Positive Calibrator cutoff (RLU ratio of 1 or more), as specified in the *digene* HC2 assay instructions.

Sensitivity, specificity, and accuracy of the hr- and lrHPV portions of the vaginal health assay were evaluated using the *digene* HC2 HPV DNA assay as the gold standard, and extracted DNA from 601 (hrHPV) and 148 (lrHPV) vaginal swabs, respectively, as the input for both tests. The vaginal health assay was considered to be positive for a HPV type if the number of reads assigned to that HPV type divided by the normalized number of reads assigned to a spiked-in control (see above) was greater than 0.1. Agreement between the two methods was evaluated using Cohen’s kappa [57], where the level of agreement is defined by the range: 0–0.20, poor; 0.21–0.40, fair; 0.41–0.60, moderate; 0.61–0.80, good; 0.81–1.00, very good.

### Sequence availability

Bacterial sequence reads of the 31 bacterial taxa and 19 HPV strains targeted by our assay are available at the European Nucleotide Archive under accession number PRJEB25853 for all human samples as well as pools of human samples reported in this article.

## Results

### Limit of detection of bacterial and HPV targets

**Fig 2:**
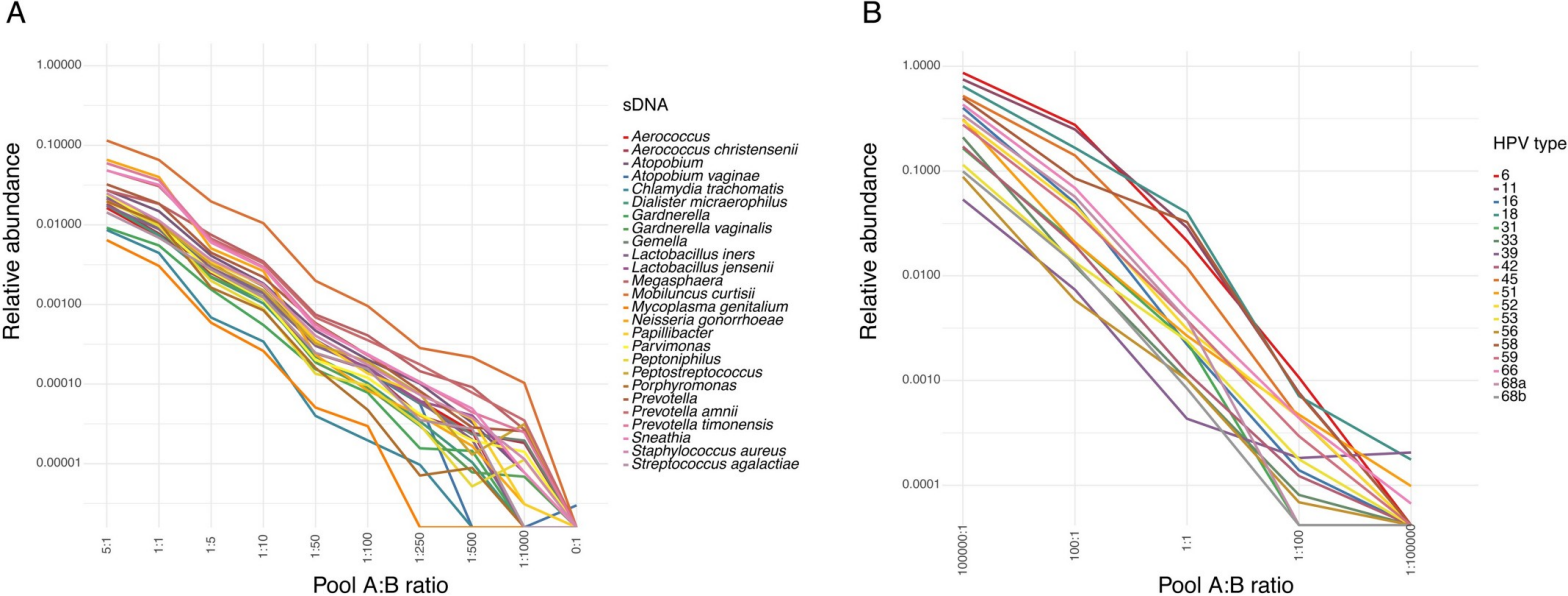
Limit of detection (LOD) of sDNAs representing bacterial and viral targets in a complex background of other sDNAs. Dilutions of two pools of sDNAs were mixed in different amounts, and microbial targets were amplified and sequenced. For each dilution and target, the relative abundance in samples with 10,000 reads or more are shown. **A.** LOD of bacterial targets. LOD read thresholds are provided in S6 Table. **B.** LOD of HPV targets. Two different sDNAs were used to represent hrHPV type 68. For each dilution and HPV type, the relative abundance in samples with 10,000 reads or more are shown. The LOD read thresholds for each HPV target are provided in S7 Table.

The vaginal health assay described here is based on a list of 31 bacterial 16S rRNA gene targets and 19 HPV types that were identified through an exhaustive literature search to play important roles in health and disease of women’s reproductive tracts (Fig 1, S1 Table). For each bacterial target, the LOD was determined by combining different dilutions of pools of sDNAs, followed by DNA extraction, amplification of the V4 region of the 16S rRNA gene using broad-range primers, and sequencing (Fig 2A). The LOB was set as the average number of reads in 77 blank wells (18.57 reads) plus 1.65 standard deviations (29.70 reads). Using this value, we calculated the threshold of identification for each taxon as the LOB + 1.65 standard deviations (48.27) plus the standard deviation of the taxon at LOD * 1.65. For the 31 taxa targeted by the assay, the threshold related to LODs was in the range 49.0 to 59.1reads (S6 Table).

To determine the LOD for the HPV targets, different dilutions of pools of sDNAs were mixed as done for the bacterial targets. The molecules were then amplified, sequenced, and analyzed by the HPV bioinformatics pipeline. For all HPV targets analyzed, the threshold related to LODs was in the range 40.8 to 224.8 reads (Fig 2B, S7 Table).

### Intra- and inter-run variability

**Fig 3:**
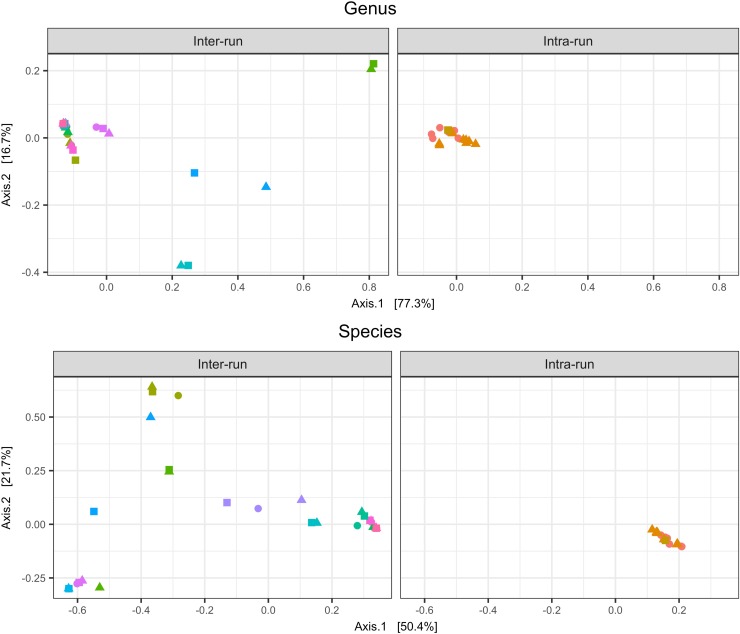
Inter- and intra-run reproducibility. PCoA ordination showing clustering of inter-run (11 vaginal samples analyzed in triplicate on three independent sequencing runs) and intra-run (18 aliquots of the same vaginal pool) data, at the genus and species taxonomic level. Shapes indicate the sequencing run, while colors indicate sample replicates. For each category (Species and Genus) samples were processed together, sharing the same scale in the visualization.

Intra-run technical variability was evaluated in a combined set of 18 replicates of the same vaginal pool, each of which yielded 10,000 reads or more. Ordination plots of both genus and species level bacterial taxa (Fig 3) showed a tight clustering of intra-run technical replicates, indicating that within a single sequencing run, results generated by the laboratory process and the bioinformatics analysis were consistent.

For the inter-run analysis, a total set of 11 groups of replicates (at least two samples) passed the filtering criteria (over 10,000 reads). The PCoA visualization at genus and species level showed a dispersion of the different samples, but with a clustering according to the respective replicates (Fig 3). This suggests that there is limited within-sample variation when the same samples are processed on different days by different operators.

### Relative abundance of bacterial targets in healthy vaginal samples

**Fig 4:**
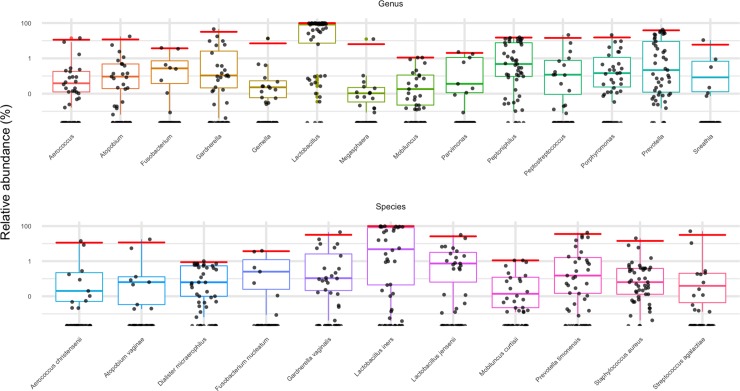
Reference ranges for the bacterial targets in the assay. A set of 50 vaginal samples, each from a different woman, was selected based on the self-reported answers given to survey questions indicating general and vaginal health. Each dot represents the relative abundance of a different bacterial target on genus level (top) or species level (bottom) within a different vaginal sample. Boxes indicate the 25th-76th percentile, with the median indicated inside each colored box. Red lines indicate the 99% percentile of each distribution, and are also the cutoff for the reference ranges. Not all of the taxa used in the assay were plotted, as some had no abundance values for this healthy cohort (*Papillibacter*, *C*. *trachomatis*, *M*. *mulieris*, *M*. *genitalium*, *N*. *gonorrhoeae*, *P*. *amnii*), based on the exclusion criteria.

To determine reference ranges for the 31 bacterial targets in the assay, we selected a set of 50 vaginal samples from our database. These represent self-reported healthy individuals from the uBiome microbiome research study. In addition to health status, additional selection criteria included no usage of antibiotics six months prior, and no current urinary tract or vaginal infections, including the presence of STDs. The 50 samples were processed with the target database, and the relative abundance ranges for each bacterial target in the cohort are shown (Fig 4).

As expected, given the nature of the samples, *Lactobacillus* was the most abundant genus, with the widest abundance distribution. At the species level, a similar distribution of the relative abundances was found, including a wide range and a high relative abundance for *Lactobacillus iners*.

### Pathogen detection

**Fig 5:**
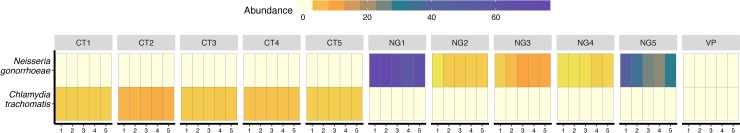
Experimental validation of 16S rRNA gene sequencing for pathogen detection using verification samples. Ten de-identified clinical verification specimens (iSpecimen) containing either *C*. *trachomatis* (n = 5) or *N*. *gonorrhoeae* (n = 5), as well as a vaginal pool (VP) constructed by combining 96 vaginal samples from 11 individuals, were tested for the presence of either pathogen using 16S rRNA gene amplification and sequencing. Five replicates of each specimen were tested. The heatmap shows the relative abundance of the two pathogens in each replicate experiment, on a scale from light yellow (absent) to dark purple (100% relative abundance).

Among the 31 bacterial targets in the assay are three pathogens implicated in STI: *C*. *trachomatis*, *N*. *gonorrhoeae*, and *M*. *genitalium*. The performance of the assay to detect two of these pathogens was confirmed on a set of ten clinical samples available through a commercial source, five of which were positive for *C*. *trachomatis*, and five of which were positive for *N*. *gonorrhoeae*. A vaginal pool consisting of samples derived from 11 healthy individuals was included as a control sample, and was found to be negative (Fig 5).

The three STI-associated targets (*C*. *trachomatis*, *M*. *genitalium*, and *N*. *gonorrhoeae*) were not present in any of the 50 samples from the healthy subject set (see also below), nor in a set of 87 vaginal samples used to validate the performance of the *digene* test on extracted DNA (see below). Twelve of thirteen positive *M*. *genitalium* samples found in a larger set of samples used to compare the HPV genotyping part of the assay (see below) were confirmed to be positive in an *M*. *genitalium* specific adhesin PCR described by others [43], while eight out of nine negative samples were confirmed to be negative, corresponding to a sensitivity of 92.3% and a specificity of 88.9% (not shown).

### Performance of *digene* HC2 HPV test on extracted DNA

**Fig 6:**
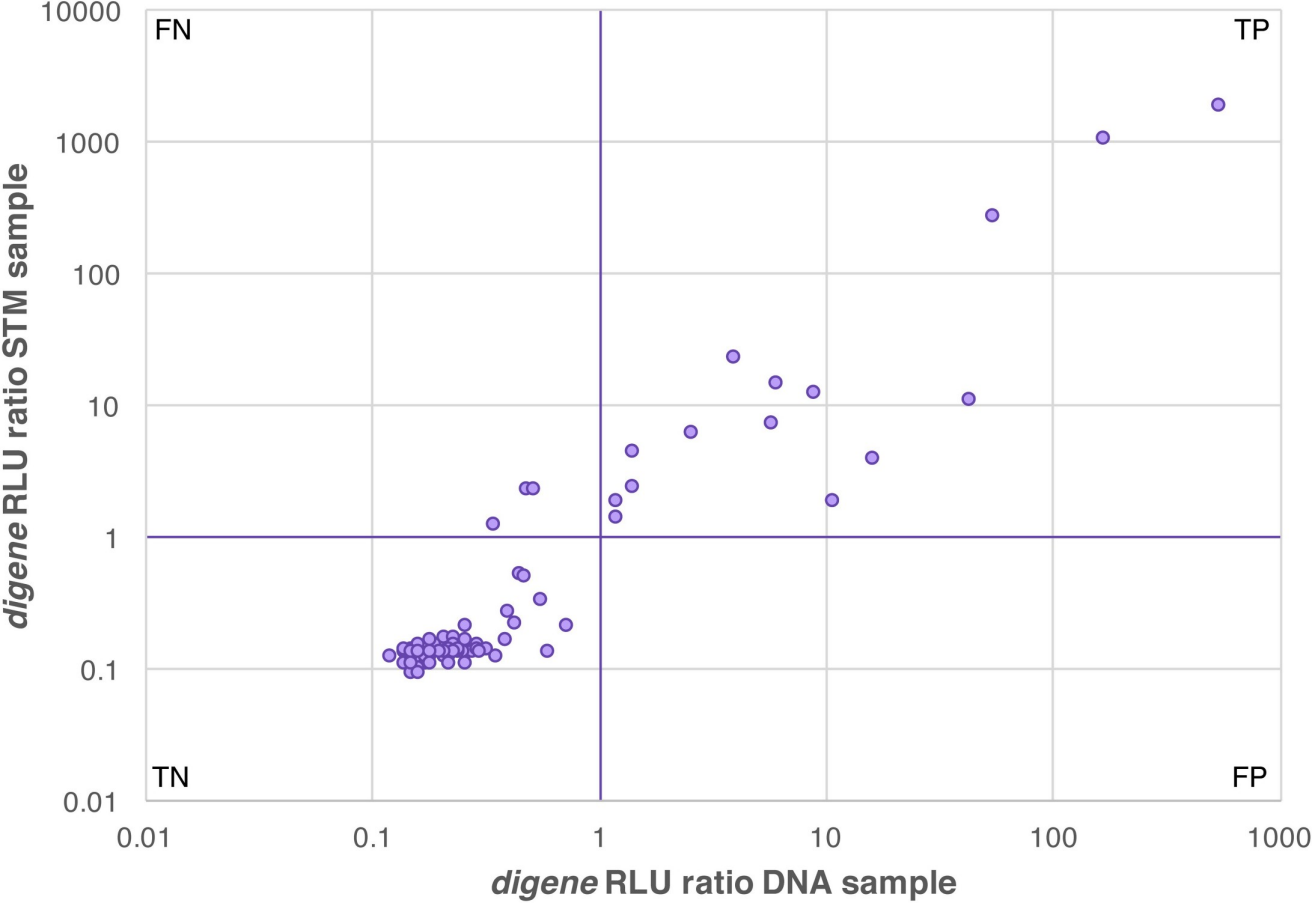
*digene* HC2 High-Risk HPV assay performance on a set of 87 paired, self-collected cervicovaginal samples. Samples were tested directly from STM tubes or from a paired sample after DNA extraction. The purple lines show the cutoff of the *digene* assay (RLU ratio = 1). Three STM specimens were positive for hrHPV with an average RLU ratio of <2 (low positive), but below RLU ratio = 1 for their corresponding extracted DNA specimen. The results for all other 84 specimens were concordant. TN, true negative; TP, true positive; FN, false negative; FP, false positive.

In order to validate the use of the *digene* kit on extracted vaginal DNA, we compared the performance of the *digene* HC2 HPV assay on a set of 87 self-obtained, paired cervicovaginal samples, i.e. a *digene* brush resuspended in *digene* STM, as well as DNA extracted from a paired vaginal swab resuspended in lysis transport medium. Of the 87 samples, 84 showed concordant results (69 were negative and 15 were positive for HPV in both tests) (Table 1). Three sample pairs that were positive with the *digene* STM sample were HPV negative when the corresponding test was performed on extracted DNA. These three samples had an average *digene* RLU ratio of 1.94, suggesting that these contained low levels of HPV (Fig 6). Agreement between *digene* STM and *digene* DNA was of 96.6%, with a sensitivity of 83.3%, a specificity of 100%, and a Cohen’s Kappa of 0.89 ± 0.12 (Z = 8.39, p-value = 0.0001).

**Table 1:** *digene* HC2 High-Risk HPV assay performance on a set of 87 paired, self-collected vaginal samples. One set of samples was collected using a *digene* brush resuspended in *digene* Specimen Transport medium (“*digene* STM”), and the second set was extracted DNA from swabs suspended in tubes with lysis/stabilization buffer (“*digene* DNA”). Samples were considered to be HPV positive if the RLU ratio was 1 or more, as instructed in the *digene* protocol.

	*digene* STM hrHPV +	*digene* STM hrHPV -	Sum
***digene* DNA hrHPV +**	15	0	**15**
***digene* DNA hrHPV -**	3	69	**72**
**Sum**	**18**	**69**	**87**

### Performance of the hrHPV sequencing test on clinical samples

Using 601 vaginal specimens, the performance of the assay to detect hrHPV was compared to that of the *digene* HC2 hrHPV assay. Of the 601 samples, 504 were negative in both tests, while 69 were positive in both tests (Table 2). Unexpectedly, three samples that were positive by sequencing for hrHPV 66, which is not covered by the *digene* hrHPV probe, were still positive in the *digene* assay. Ten *digene*-positive samples did not yield any validated hrHPV reads after amplification and sequencing by our assay. Of these ten false negatives, six samples were found to contain single or mixed non-high risk HPV strains by genotyping, including HPV 30, 61, 40, 42, 53, and 67. In addition, eighteen samples were negative in the *digene* HC2 hrHPV assay but yielded sufficient hrHPV reads (of types 16, 31, 35, 51, 52, 56, 59, and 68b) to be identified as positives by our genotyping assay. Thus, in comparison to the *digene* HC2 hrHPV test, the hrHPV sequencing assay had a sensitivity of 87.3% and a specificity of 96.6%, with an overall agreement of 95.3%, and a Cohen’s kappa of 0.804 ± 0.070 (Z = 19.8; p-value <0.0001). After removal of the six samples where cross-reactivity of the digene hrHPV probe with lrHPV sequences was suspected, the sensitivity and specificity of the hrHPV genotyping assay were 94.5% and 96.6%, respectively, with a kappa of 0.841 ± 0.065.

**Table 2:** Comparison of the vaginal health assay for the detection of genotyped hrHPV in DNA extracted from 601 vaginal samples to that of the *digene* HC2 hrHPV assay. The genotyping assay was considered positive if the normalized number of reads assigned to any of the validated hrHPV types (16, 18, 31, 33, 35, 39, 45, 51, 52, 56, 58, 59, 66, and 68) divided by the number of reads assigned to a spike in control was greater than 0.1. The *digene* test was considered positive if the measured RLU was equal to or greater than the assay’s cutoff (RLU ratio of 1 or higher), as per the manufacturer’s instructions.

	*digene* hrHPV +	*digene* hrHPV -	Sum
**Genotyping** **hrHPV +**	69	18	87
**Genotyping** **hrHPV -**	10	504	514
**Sum**	79	522	601

**Fig 7:**
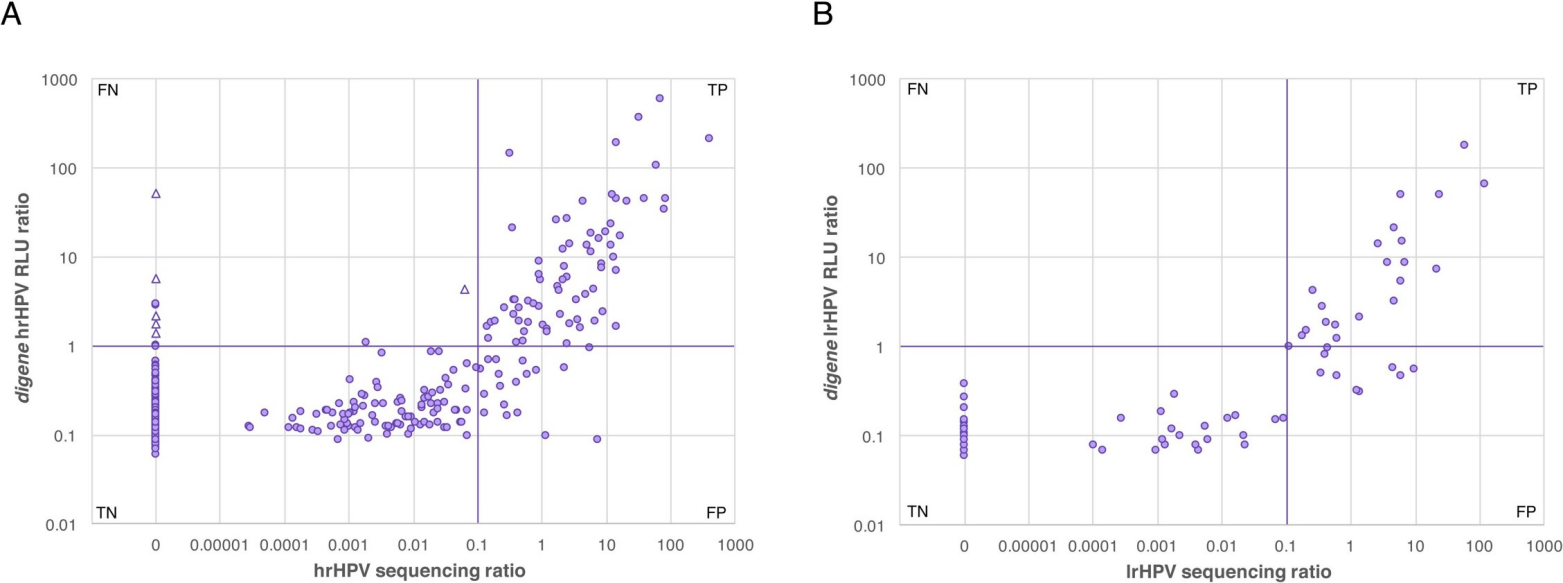
Comparison of hrHPV and lrHPV amplification and genotyping to the *digene* HC2 HPV assay. DNA from 718 vaginal samples was extracted and tested by PCR amplification and sequencing using HPV primers, and additionally used directly in the *digene* assay using the HC2 hrHPV (panel A) or lrHPV (panel B) probe mix. For each sample, the x-axis shows the normalized ratio of reads assigned to validated HPV types over reads assigned to a spiked-in internal control, while the Y-axis shows the *digene* HPV probe RLU values normalized over the assay’s cut-off RLU. The two purple lines show the cutoff for each of the assays. **A.** Comparison of hrHPV test results in a subset of 601 samples. Six samples that were positive in the *digene* hrHPV assay and negative in the hrHPV genotyping assay, but in which lrHPV sequences were detected by genotyping, are shown as light purple triangles. **B.** Comparison of lrHPV test results in a subset of 148 samples.

Good correlation was found between the number of normalized hrHPV sequencing reads and the *digene* HC2 hrHPV RLU ratios, confirming that the PCR and sequencing hrHPV assay described here can not only detect hrHPV types but also assess their relative abundance (Fig 7A).

### Performance of the lrHPV sequencing test on clinical samples

Using 148 vaginal specimens, the performance of the assay to detect lrHPV by genotyping was compared to that of the *digene* HC2 lrHPV assay. Of the 148 samples, 118 were negative in both tests, while 21 were positive in both tests (Table 3). No false negatives were found, but nine samples that were negative in the *digene* lrHPV assay were found to have lrHPV sequences by genotyping, of types 42, 43, and 44. Using the *digene* HC2 lrHPV test as the gold standard, the lrHPV genotyping assay was found to have a sensitivity of 100% and a specificity of 92.9%, with an overall agreement of 93.9%, Cohen’s kappa = 0.788 ± 0.131 (Z = 9.81, p-value < 0.0001).

**Table 3:** Comparison of the vaginal health assay for the detection of lrHPV in DNA extracted from 148 vaginal samples by genotyping to that of the *digene* HC2 lrHPV assay. The genotyping assay was considered positive if the normalized number of reads assigned to any of the validated lrHPV types (6, 11, 42, 43, 44) divided by the number of reads assigned to a spike in control was greater than 0.1. The *digene* test was considered positive if the measured RLU was equal to or greater than the assay’s cutoff (RLU ratio of 1 or higher), as per the manufacturer’s instructions.

	*digene* lrHPV +	*digene* lrHPV -	Sum
**Genotyping** **lrHPV +**	21	9	30
**Genotyping** **lrHPV -**	0	118	118
**Sum**	21	127	148

As with the hrHPV assay, the number of normalized lrHPV sequencing reads was positively correlated to the *digene* HC2 lrHPV RLU ratios, confirming that the PCR and sequencing lrHPV assay described here can not only detect lrHPV types but also assess their relative abundance (Fig 7B).

### Positive clinical samples for hrHPV and lrHPV types

**Fig 8:**
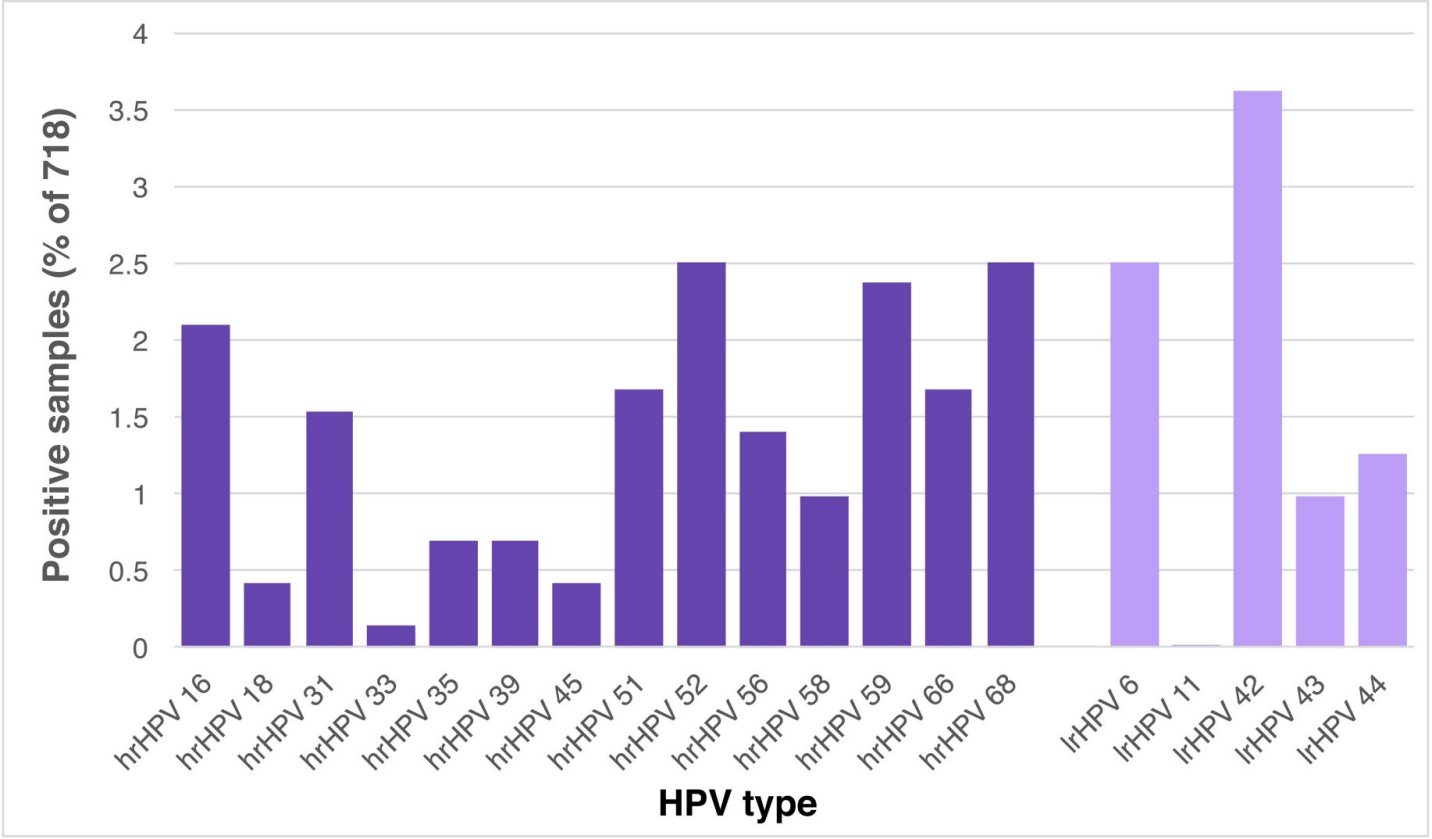
Distribution of the 19 HPV types in this study. A set of 718 vaginal samples was tested in the HPV genotyping assay described in this study. Of these, 142 samples were positive for at least one of the 19 HPV types validated in this study, with 100 samples positive for hrHPV, and 60 for lrHPV. In 35 of the 142 positive samples, two or more HPV types were found. Positive samples are plotted as percentage found in the total set of 718 samples.

In the combined 718 samples used in the comparison of the vaginal health assay to the *digene* HC2 HPV assay, 142 samples were found to be positive for 1 or more of the 19 HPV types. Of these, 107 samples contained only a single HPV type, while 35 samples contained 2 or more HPV types. Among the 100 samples that contained at least one of the 14 validated hrHPV types, HPV types 52 (13%), 68 (13%), 59 (12%), and 16 (11%) were the most common. Within the 60 samples positive for at least one of the 5 validated lrHPV types, HPV type 42 (43%) and 6 (30%) were the most common, while type 11 was not found (Fig 8).

## Discussion

Here, we describe a novel vaginal health assay combining vaginal microbiome analysis, STI-associated pathogen detection, and HPV detection and identification in a self-sampling format. Although each of these components have been described before, to our knowledge, this assay is the first to combine all of these parts, thus offering women a unique opportunity to gain a broad perspective into their vaginal and reproductive health.

The detection of hrHPV in combination with vaginal self-sampling has been proposed as an effective method for cervical cancer risk screening [58]. Although the sensitivity of probe hybridization-based hrHPV detection, such as the *digene* HC2 assay, in self-obtained vaginal swabs has been found to be slightly lower than that in clinician-obtained cervical specimens, hrHPV detection based on PCR was shown to be equally sensitive in self-sampled specimens [31]. While the vaginal health assay described in this study is not intended to replace regular cervical cancer screening programs, offering women the opportunity to self-collect vaginal specimens poses fewer barriers for women to be screened, and thus could lead to increased participation rates [59,60]. Therefore, allowing self-collection of vaginal samples for hrHPV screening in parallel to regular screening programs, as already implemented in numerous countries, and recommending women to seek further physician examination in case of a positive result may have a positive impact on rates of detection of cervical cancer, and potentially save lives [58].

The vaginal health assay described here not only detects whether HPV is present in a sample, but also identifies the presence of specific type(s) by using sequencing analysis. Several other HPV genotyping assays based on PCR and sequencing have been reported [30, 61–65]. These studies detected HPV types not found by traditional methods [62], as well as infections with multiple types [63–65] with high sensitivity. As recommended by the VALGENT study framework [66], we compared the performance of the HPV component of the test to that of the widely used *digene* HC2 HPV assay. Because the novel test reported here is performed on extracted DNA, we first validated the use of the *digene* assay on extracted DNA. The *digene* performance on the extracted DNA was slightly less sensitive than that directly performed on the *digene* STM tubes. The *digene* HC2 assay has been found to give discordant results in about 8% of paired tests [67,68], where, for example, a positive sample will test negative at retesting, most often in samples with a low RLU ratio in the positive test. A cutoff ratio of 2 or 3 instead of 1 has been proposed to serve as a better indicator for reproducible positive results [67–70]. In our study, all specimens with RLU ratios of 2 or higher in the direct *digene* HC2 test on STM tubes were also correctly identified as positive when the test was performed on extracted DNA, suggesting that the *digene* assay can be applied to extracted DNA as well.

Using extracted DNA from 718 vaginal specimens as the template, the performance of the HPV genotyping parts of the vaginal health assay was compared to that of the *digene* HC2 HPV assay. The hrHPV genotyping assay showed good correlation with the *digene* assay, with a sensitivity and specificity of 87.3% and 96.6%, respectively, and a Cohen’s kappa of 0.804. Three samples containing hrHPV type 66 were found to be positive in both tests, even though they are supposedly not covered by the *digene* assay. Among a set of ten samples that were reported positive by the hrHPV *digene* assay but that did not contain hrHPV sequences as determined by our assay, six samples were found to contain single or mixed lrHPV types, including HPV 30, 61, 40, 42, 53, and 67. Cross-reactivity of the *digene* HC2 hrHPV probe mix with lrHPV sequences has been demonstrated by several others [69,71–74]. Thus, even though these samples had to be classified as false-negative because the *digene* HC2 HPV assay was taken as the gold standard, it is likely they were actually false-positives in the *digene* test. Removing these six samples from the comparison, the sensitivity and specificity of the hrHPV genotyping assay were 94.5% and 96.6%, respectively, with a kappa of 0.841 ± 0.065. The lrHPV genotyping assay had a sensitivity of 100% and a specificity of 92.9%, with a Cohen’s kappa of 0.788. In addition, the two tests were in good general agreement about the relative amount of HPV molecules detected. The specific genotyping of HPV by sequencing therefore can detect the presence of lrHPV that are known to give false-positive results in conventional clinical hrHPV tests. In addition, HPV genotyping provides clinicians and patients with more detailed information about which strain(s) are present in the vagina, allowing for more precise tracking of infection and clearance than most conventional assays [30].

Among the samples tested positive with hrHPV genotyping, HPV types 16, 52, 59, and 68 were most common. HPV types 6 and 42 were the most prevalent lrHPV types in the samples tested in this study. Although our sample set was small (718 samples, of which 142 tested positive) and only screened for the presence of 19 HPV types, the high proportion of positive samples for types 6, 16, 52, 59 has been found by others [75,76], while the results for hrHPV type 68 appears to be unique in our dataset.

In addition to the HPV portion of the novel vaginal health assay described here, the assay also reports the relative abundance of commensal and pathogenic bacteria in vaginal samples, and compares these to reference ranges. Self-collection has been shown to be well-suited for vaginal microbiome analysis as reported by Forney et al. who showed that microbial diversity is similar between self-collected and physician collected vaginal samples [77].

Several bacteria have been associated with vaginal health conditions, such as bacterial vaginosis [1,4–6], aerobic vaginitis [7], pelvic inflammatory disease [44], and sexually transmitted infections [9–12]. The vaginal health assay described here detects the relative abundance of bacteria positively associated with bacterial vaginosis, such as *Sneathia* or *Gardnerella* species, as well as those negatively associated with that condition such as *Lactobacillus* species. In addition, it detects the presence of three common STI-associated pathogens, i.e., *C*. *trachomatis*, *N*. *gonorrhoeae*, and *M*. *genitalium*. Of these, *M*. *genitalium* has been recently recognized as an important pathogen implicated in pelvic inflammatory disease and infertility [12,78]. Although some early diagnostic tests have been described [79,80], very few clinicians test for its presence. Furthermore, the vaginal microbiota composition has been reported to be associated with the progression of HPV infection, from early states to cervical cancer [37,40]. Vaginal microbiome analysis therefore not only can be used to detect STI-associated pathogens and bacteria involved in bacterial vaginosis, but also to assess a woman’s microbiome similarity to the microbiome of a group of individuals with progressed HPV infection. This brings about the opportunity to leverage microbiome information to understand HPV infection progression and women's susceptibility to cancer development. Future versions of this assay could also include additional microorganisms associated with pathogenic outcomes such as *Trichomonas vaginalis*, *Mycoplasma parvum*, and *Ureaplasma urealithicum*.

In conclusion, we here present a vaginal health assay that for the first time combines the detection of the most important bacterial and viral indicators of vaginal health and disease. We envision that this test has the potential to provide clinicians and patients with a more comprehensive understanding of the vaginal microbiome, and to encourage women to take an active role in related conversations with their doctors. We also hope that by improving accessibility through self-sampling, we may encourage more women to engage with current screening and treatment guidelines for vaginal pathogens and cervical cancer.

## Supporting information

S1 Fig Vaginal sampling instructions. Participants in this study were sent a vaginal sampling kit containing a swab, sterile water to pre-wet the swab, a tube containing zirconia beads and a lysis and stabilization buffer, and sampling instructions such as the one shown above. After sampling according to the instructions, participants could ship their sample back by regular mail.
(PDF)

S2 Fig *In silico* performance metrics of the bacterial targets. Initially, 72 bacterial targets were identified based on their association with vaginal and reproductive health, comprised of 57 species and 15 genera. The following performance metrics were evaluated based on the number of true positives (TP), true negatives (TN), false positives (FP), and false negatives (FN) detected in a manually curated amplicon database (described in S1 Doc in Almonacid *et al*., 2017). The target performance are plotted as follows: specificity = TN / (TN + FP); sensitivity = TP / (TP + FN); positive predictive value (PPV) = TP / (TP + FP); and negative predictive value (NPV) = TN / (TN + FN). Based on a cutoff of 90% (red vertical line), 31/72 preliminary targets passed for each of the parameters, resulting in the accurate *in silico* detection of 16 bacterial species (light purple), and 15 bacterial genera (dark purple).
(PDF)

S1 Table Assay targets and associated health conditions. List of all 31 bacterial targets and 19 HPV targets, their associations with different health conditions, and references.
(PDF)

S2 Table *In silico* performance metrics for the 72 bacterial targets (genus and species level) that were initially selected. PPV, positive predictive value; NPV, negative predictive value. All values in the sensitivity, specificity, PPV, and NPV columns are given as percentages. Of the 72 selected targets, 31 passed selection criteria of all values above 90%. Values below 90% are shown in red.
(PDF)

S3 Table *In silico* performance metrics for the 19 HPV targets. HPV, human papillomavirus; TP, true positive; FN, false negative; FP, false positive; TN, true negative; Sens, sensitivity (in %); Spec, specificity (in %); PPV, positive predictive value (in %); NPV, negative predictive value (in %).
(PDF)

S4 Table List of the 31 synthetic DNAs created to represent the bacterial targets included in the assay. Sequences are based on the 16S rRNA gene.
(PDF)

S5 Table List of the 20 synthetic DNA sequences representing 5 lrHPV and 14 hrHPV types included in the assay. Because of sequence variability, hrHPV type 68 was represented by 2 different sDNAs.
(PDF)

S6 Table Limit of detection (LOD) assay for the bacterial targets. The table shows the lowest dilution at which at least two of the three replicates had 2 or more reads per taxon, and the calculated threshold for identification per taxa at the LOD in number of reads.
(PDF)

S7 Table Limit of detection (LOD) assay for the HPV targets. The table shows the lowest dilution at which at least two of the three replicates had 2 or more reads per HPV type, and the calculated threshold for identification per HPV type at the LOD in number of reads.
(PDF)
