# Supplementary material for: A novel sequencing-based vaginal health assay combining self-sampling, HPV detection and genotyping, STI detection, and vaginal microbiome analysis
Source: PLoS One. 2019 May 1;14(5):e0215945. doi: 10.1371/journal.pone.0215945 (PMC6493738; doi:10.1371/journal.pone.0215945)
Supplement: S1 Fig — Participants in this study were sent a vaginal sampling kit containing a swab, sterile water to pre-wet the swab, a tube containing zirconia beads and a lysis and stabilization buffer, and sampling instructions such as the one shown above. After sampling according to the instructions, participants could ship their sample back by regular mail. (PDF) [file pone.0215945.s001.pdf]

Supplementary material belonging to

“A novel sequencing-based vaginal health assay combining self-sampling, HPV detection and genotyping, STI detection, and vaginal microbiome analysis”

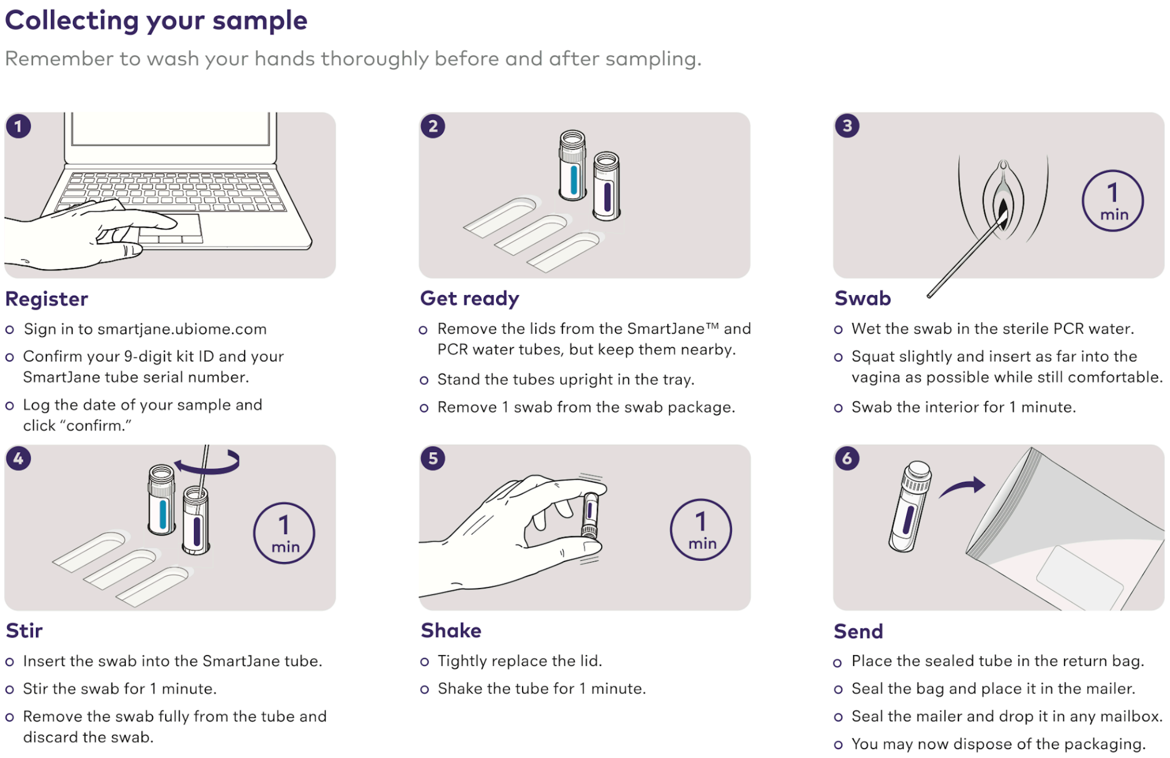

S1 Figure. Vaginal sampling instructions. Participants in this study were sent a vaginal sampling kit containing a swab, sterile water to pre-wet the swab, a tube containing zirconia beads and a lysis and stabilization buffer, and sampling instructions such as the one shown above. After sampling according to the instructions, participants could ship their sample back by regular mail.
