# Supplementary material for: A novel sequencing-based vaginal health assay combining self-sampling, HPV detection and genotyping, STI detection, and vaginal microbiome analysis
Source: PLoS One. 2019 May 1;14(5):e0215945. doi: 10.1371/journal.pone.0215945 (PMC6493738; doi:10.1371/journal.pone.0215945)
Supplement: S2 Fig — Initially, 72 bacterial targets were identified based on their association with vaginal and reproductive health, comprised of 57 species and 15 genera. The following performance metrics were evaluated based on the number of true positives (TP), true negatives (TN), false positives (FP), and false negatives (FN) detected in a manually curated amplicon database (described in S1 Doc in Almonacid et al., 2017). The target performance are plotted as follows: specificity = TN / (TN + FP); sensitivity = TP / (TP + FN); positive predictive value (PPV) = TP / (TP + FP); and negative predictive value (NPV) = TN / (TN + FN). Based on a cutoff of 90% (red vertical line), 31/72 preliminary targets passed for each of the parameters, resulting in the accurate in silico detection of 16 bacterial species (light purple), and 15 bacterial genera (dark purple). (PDF) [file pone.0215945.s002.pdf]

Supplementary material belonging to

"A novel sequencing-based vaginal health assay combining self-sampling, HPV detection and genotyping, STI detection, and vaginal microbiome analysis"

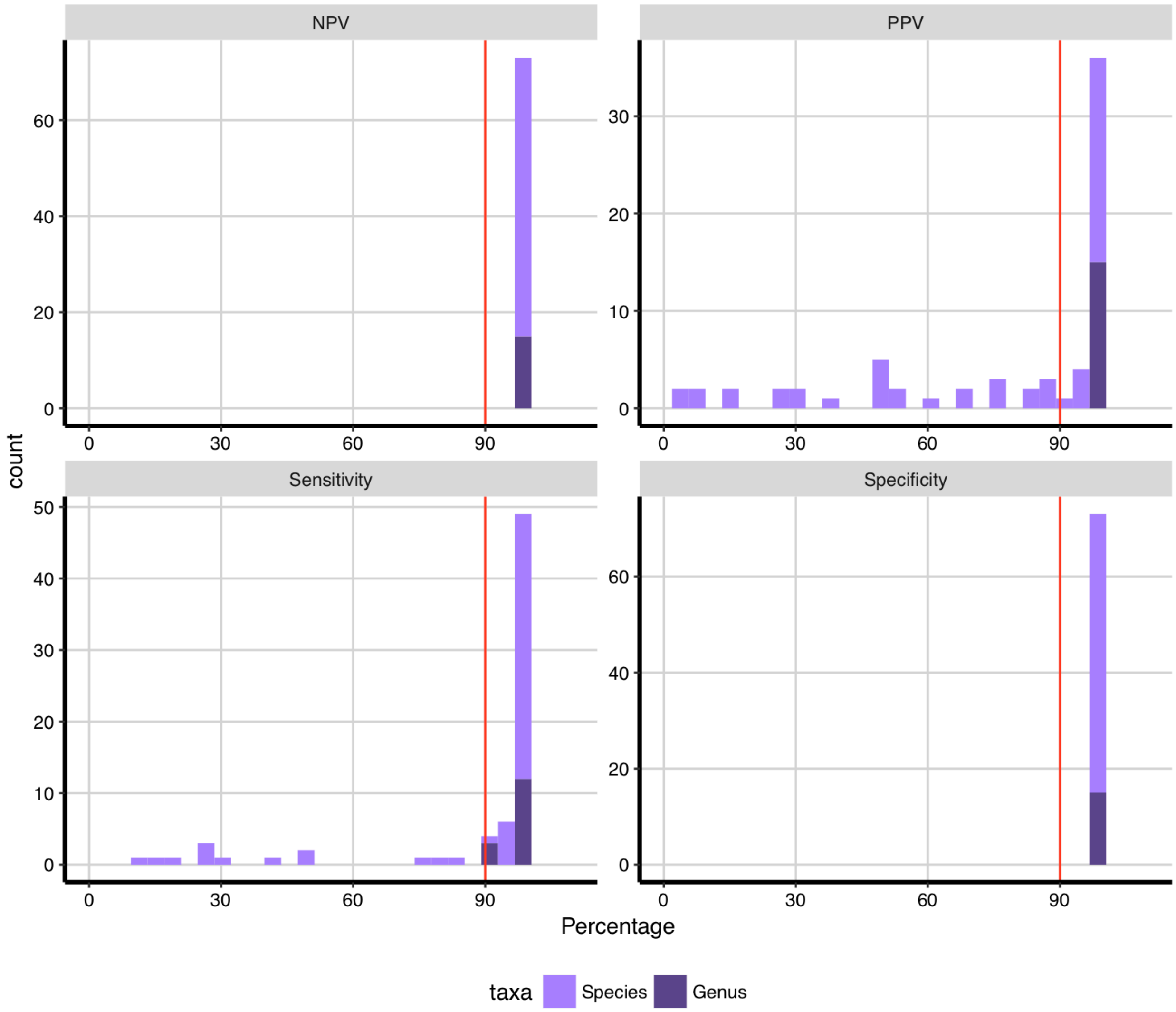

S2 Figure. *In silico* performance metrics of the bacterial targets. Initially, 72 bacterial targets were identified based on their association with vaginal and reproductive health, comprised of 57 species and 15 genera. The following performance metrics were evaluated based on the number of true positives (TP), true negatives (TN), false positives (FP), and false negatives (FN) detected in a manually curated amplicon database (described in S1 Doc in Almonacid *et al*., 2017). The target performance are plotted as follows:

specificity = TN / (TN + FP); sensitivity = TP / (TP + FN); positive predictive value (PPV) = TP / (TP + FP); and negative predictive value (NPV) = TN / (TN + FN). Based on a cutoff of 90% (red vertical line), 31/72 preliminary targets passed for each of the parameters, resulting in the accurate *in silico* detection of 16 bacterial species (light purple), and 15 bacterial genera (dark purple).
