# Supplementary material for: A novel sequencing-based vaginal health assay combining self-sampling, HPV detection and genotyping, STI detection, and vaginal microbiome analysis
Source: PLoS One. 2019 May 1;14(5):e0215945. doi: 10.1371/journal.pone.0215945 (PMC6493738; doi:10.1371/journal.pone.0215945)
Supplement: S1 Table — List of all 31 bacterial targets and 19 HPV targets, their associations with different health conditions, and references. (PDF) [file pone.0215945.s003.pdf]

Supplementary material belonging to

“A novel sequencing-based vaginal health assay combining self-sampling, HPV detection and genotyping, STI detection, and vaginal microbiome analysis”

S1 Table. **Assay targets and associated health conditions.** List of all 31 bacterial targets and 19 HPV targets, their associations with different health conditions, and references.

Target	Taxonomic rank	Association	Health condition	References
Aerococcus	genus	Associated	Bacterial Vaginosis	Ling et al., 2010; Ravel et al., 2011
Aerococcus christensenii	species	Associated	Bacterial Vaginosis	Srinivasan et al., 2012
Atopobium	genus	Associated	Bacterial Vaginosis	Biagi et al., 2009; Ravel et al., 2011
Atopobium vaginae	species	Associated	Bacterial Vaginosis	Ling et al., 2010; Srinivasan et al., 2012; Datcu et al., 2013
Chlamydia trachomatis	species	Associated	Sexually Transmitted Infection	Lane and Decker, 2016; Ziklo et al., 2016; Budrys et al., 2012
	species	Associated	Cervicitis	Dehon and McGowin, 2014
	species	Associated	Pelvic Inflammatory Disease	Brunham et al., 2015; Jossens et al., 1994; De Muylder et al., 1990
	species	Associated	Idiopathic Infertility	De Muylder et al., 1990
Dialister micraerophilus	species	Associated	Bacterial Vaginosis	Srinivasan et al., 2012; Xia et al., 2016
Fusobacterium	genus	Associated	Bacterial Vaginosis	Ravel et al., 2011

Fusobacterium nucleatum	species	Inversely associated	Human papillomavirus Infection	Shannon et al., 2017
Gardnerella	genus	Associated	Bacterial Vaginosis	Ravel et al., 2011
	genus	Associated	Human papillomavirus Infection	Murta et al., 2000
Gardnerella vaginalis	species	Associated	Bacterial Vaginosis	Ling et al., 2010; Srinivasan et al., 2012; Biagi et al., 2009; Delaney and Onderdonk, 2001
	species	Associated	Aerobic Vaginitis	Donders et al., 2005
	species	Associated	Human papillomavirus Infection	Gao et al., 2013
Gemella	genus	Associated	Bacterial Vaginosis	Ravel et al., 2011; Xia et al., 2016
Lactobacillus	genus	Inversely associated	Cervicitis	Anahtar et al., 2015
	genus	Inversely associated	Bacterial Vaginosis	Ling et al., 2010; Srinivasan et al., 2012
Lactobacillus iners	species	Inversely associated	Bacterial Vaginosis	Ling et al., 2010; Ravel et al., 2011; Srinivasan et al., 2012
	species	Inversely associated	Human papillomavirus Infection	Lee et al., 2013
Lactobacillus jensenii	species	Inversely associated	Bacterial Vaginosis	Ravel et al., 2011; Srinivasan et al., 2012; Fettweis et al., 2014
Megasphaera	genus	Associated	Bacterial Vaginosis	Ling et al., 2010; Ravel et al., 2011; Srinivasan et al., 2012
Mobiluncus	genus	Associated	Bacterial Vaginosis	Ravel et al., 2011
Mobiluncus curtisii	species	Associated	Bacterial Vaginosis	Schwebke and Lawing, 2001; Meltzer et al., 2008

Mobiluncus mulieris	species	Associated	Bacterial Vaginosis	Srinivasan et al., 2012; Datcu et al., 2013
Mycoplasma genitalium	species	Associated	Sexually Transmitted Infection	Jensen, 2017
	species	Associated	Cervicitis	Dehon and McGowin, 2014
	species	Associated	Infertility	Grześko et al., 2009
Neisseria gonorrhoeae	species	Associated	Sexually Transmitted Infection	Hill et al., 2016
	species	Associated	Pelvic Inflammatory Disease	Brunham et al., 2015; Jossens et al., 1994; De Muylder et al., 1990; Soper et al., 1994; Hebb et al, 2004; Brunham et al., 1988
	species	Associated	Infertility	De Muylder et al., 1990
Papillibacter	genus	Associated	Bacterial Vaginosis	Ling et al., 2010
Parvimonas	genus	Associated	Bacterial Vaginosis	Ravel et al., 2011
Peptoniphilus	genus	Associated	Bacterial Vaginosis	Ravel et al., 2011
Peptostreptococcus	genus	Associated	Bacterial Vaginosis	Ravel et al., 2011; Delaney and Onderdonk, 2001
Porphyromonas	genus	Associated	Bacterial Vaginosis	Ravel et al., 2011
Prevotella	genus	Associated	Bacterial Vaginosis	Ling et al., 2010; Ravel et al., 2011; Srinivasan et al., 2012; Delaney and Onderdonk, 2001; Datcu et al., 2013
Prevotella amnii	species	Associated	Bacterial Vaginosis	Srinivasan et al., 2012; Macklaim et al., 2013
Prevotella timonensis	species	Associated	Bacterial Vaginosis	Srinivasan et al., 2012; Xia et al., 2016
Sneathia	genus	Associated	Bacterial Vaginosis	Ravel et al., 2011; Srinivasan et al., 2012

	genus	Associated	Human papillomavirus Infection	Lee et al., 2013
Staphylococcus aureus	species	Associated	Aerobic Vaginitis	Donders et al., 2002; Donders et al., 2005; Donders et al., 2017
Streptococcus agalactiae	species	Associated	Aerobic Vaginitis	Donders et al., 2005; Donders et al., 2017
HPV	-	Associated	Human papillomavirus Infection	Moscicki, 2005; Inan et al., 2017; Bauer et al., 1991; Doorbar et al., 2015; Baseman and Koutski, 2005
lrHPV (types 6, 11, 42, 43, and 44)	-	Associated	Genital warts	X.-L. Wu et al., 2010, Doorbar et al., 2017
hrHPV (types 16, 18, 31, 33, 35, 39, 45, 51, 52, 56, 58, 59, 66, and 68)	-	Associated	Cervical Cancer	Dartell et al., 2014; McDonald et al., 2014; Vidal et al., 2011; De Vuyst et al., 2012; Joshi et al., 2014;
	-	Associated	SIL (High & Low)	Dartell et al., 2014; Jarboe et al., 2004; Cappiello et al., 1997; Sellors et al., 2000; Evans et al., 2006
	-	Associated	Cervicitis	Menon et al., 2016

References

- Anahtar, M.N., Byrne, E.H., Doherty, K.E., Bowman, B.A., Yamamoto, H.S., Soumillon, M., Padavattan, N., Ismail, N., Moodley, A., Sabatini, M.E., et al. (2015). Cervicovaginal Bacteria Are a Major Modulator of Host Inflammatory Responses in the Female Genital Tract. Immunity 42, 965–976.
- Baseman, J.G., and Koutsky, L.A. (2005). The epidemiology of human papillomavirus infections. J. Clin. Virol. 32 Suppl 1, S16-24.

- Bauer, H.M., Ting, Y., Greer, C.E., Chambers, J.C., Tashiro, C.J., Chimera, J., Reingold, A., and Manos, M.M. (1991). Genital human papillomavirus infection in female university students as determined by a PCR-based method. JAMA 265, 472–477.
- Biagi, E., Vitali, B., Pugliese, C., Candela, M., Donders, G.G.G., and Brigidi, P. (2009). Quantitative variations in the vaginal bacterial population associated with asymptomatic infections: a real-time polymerase chain reaction study. Eur J Clin Microbiol Infect Dis 28, 281–285.
- Brunham, R.C., Binns, B., Guijon, F., Danforth, D., Kosseim, M.L., Rand, F., McDowell, J., and Rayner, E. (1988). Etiology and Outcome of Acute Pelvic Inflammatory Disease. J Infect Dis 158, 510–517.
- Brunham, R.C., Gottlieb, S.L., and Paavonen, J. (2015). Pelvic Inflammatory Disease. New England Journal of Medicine 372, 2039–2048.
- Budrys, N.M., Gong, S., Rodgers, A.K., Wang, J., Louden, C., Shain, R., Schenken, R.S., and Zhong, G. (2012). Chlamydia trachomatis Antigens Recognized by Women With Tubal Factor Infertility, Normal Fertility, and Acute Infection. Obstet Gynecol 119, 1009–1016.
- Cappiello, G., Garbuglia, A.R., Salvi, R., Rezza, G., Giuliani, M., Pezzotti, P., Suligoi, B., Branca, M., Migliore, G., Formigoni Pomponi, D., et al. (1997). HIV infection increases the risk of squamous intra-epithelial lesions in women with HPV infection: An analysis of HPV genotypes. Int. J. Cancer 72, 982–986.
- Dartell, M.A., Rasch, V., Iftner, T., Kahesa, C., Mwaiselage, J.D., Junge, J., Gernow, A., Ejlersen, S.F., Munk, C., and Kjaer, S.K. (2014). Performance of visual inspection with acetic acid and human papillomavirus testing for detection of high-grade cervical lesions in HIV positive and HIV negative Tanzanian women. Int. J. Cancer 135, 896–904.
- Datcu, R., Gesink, D., Mulvad, G., Montgomery-Andersen, R., Rink, E., Koch, A., Ahrens, P., and Jensen, J.S. (2013). Vaginal microbiome in women from Greenland assessed by microscopy and quantitative PCR. BMC Infectious Diseases 13, 480.
- De Muylder, X., Laga, M., Thnnstedt, C., Van Dyck, E., Aelbers, G.N.M., and Piot, P. (1990). The Role of *Neisseria gonorrhoeae* and *Chlamydia trachomatis* in Pelvic Inflammatory Disease and Its Sequelae in Zimbabwe. J Infect Dis 162, 501–5058.
- De Vuyst, H., Mugo, N.R., Chung, M.H., McKenzie, K.P., Nyongesa-Malava, E., Tenet, V., Njoroge, J.W., Sakr, S.R., Meijer, C.M., Snijders, P.J.F., et al. (2012). Prevalence and determinants of human papillomavirus infection and cervical lesions in HIV-positive women in Kenya. Br J Cancer 107, 1624–1630.
- Dehon, P.M., and McGowin, C.L. (2014). *Mycoplasma genitalium* Infection Is Associated with Microscopic Signs of Cervical Inflammation in Liquid Cytology Specimens. J. Clin. Microbiol. 52, 2398–2405.
- Delaney, M.L., and Onderdonk, A.B. (2001). Nugent score related to vaginal culture in pregnant women. Obstetrics & Gynecology 98, 79–84.
- Donders, G.G.G., Vereecken, A., Bosmans, E., Dekeersmaecker, A., Salembier, G., and Spitz, B. (2002). Definition of a type of abnormal vaginal flora that is distinct from bacterial vaginosis: aerobic vaginitis. BJOG: An International Journal of Obstetrics & Gynaecology 109, 34–43.

- Donders, G.G.G., Vereecken, A., Bosmans, E., Dekeersmaecker, A., Salembier, G., and Spitz, B. (2005). Aerobic vaginitis: Abnormal vaginal flora entity that is distinct from bacterial vaginosis. International Congress Series 1279, 118–129.
- Donders, G.G.G., Bellen, G., Grinceviciene, S., Ruban, K., and Vieira-Baptista, P. (2017). Aerobic vaginitis: no longer a stranger. Research in Microbiology. In press.
- Doorbar, J., Egawa, N., Griffin, H., Kranjec, C., and Murakami, I. (2015). Human papillomavirus molecular biology and disease association. Rev. Med. Virol. 25, 2–23.
- Evans, M.F., Adamson, C.S.-C., Papillo, J.L., St. John, T.L., Leiman, G., and Cooper, K. (2006). Distribution of human papillomavirus types in ThinPrep Papanicolaou tests classified according to the Bethesda 2001 terminology and correlations with patient age and biopsy outcomes. Cancer 106, 1054–1064.
- Fettweis, J.M., Brooks, J.P., Serrano, M.G., Sheth, N.U., Girerd, P.H., Edwards, D.J., Strauss, J.F., the Vaginal Microbiome Consortium, Jefferson, K.K., and Buck, G.A. (2014). Differences in vaginal microbiome in African American women versus women of European ancestry. Microbiology 160, 2272–2282.
- Gao, W., Weng, J., Gao, Y., and Chen, X. (2013). Comparison of the vaginal microbiota diversity of women with and without human papillomavirus infection: a cross-sectional study. BMC Infectious Diseases 13, 271.
- Grześko, J., Elias, M., Mączyńska, B., Kasprzykowska, U., Tłaczała, M., and Goluda, M. (2009). Occurrence of *Mycoplasma genitalium* in fertile and infertile women. Fertility and Sterility 91, 2376–2380.
- Hebb, J.K., Cohen, C.R., Astete, S.G., Bukusi, E.A., and Totten, P.A. (2004). Detection of Novel Organisms Associated with Salpingitis, by Use of 16S rDNA Polymerase Chain Reaction. J Infect Dis 190, 2109–2120.
- Hill, S.A., Masters, T.L., and Wachter, J. Gonorrhea - an evolving disease of the new millennium. Microb Cell 3, 371–389.
- Inan, H., Wang, S., Inci, F., Baday, M., Zangar, R., Kesiraju, S., Anderson, K.S., Cunningham, B.T., and Demirci, U. (2017). Isolation, Detection, and Quantification of Cancer Biomarkers in HPV-Associated Malignancies. Sci Rep 7, 3322.
- Jarboe, E.A., Thompson, L.C., Heinz, D., McGregor, J.A., and Shroyer, K.R. (2004). Telomerase and human papillomavirus as diagnostic adjuncts for cervical dysplasia and carcinoma. Human Pathology 35, 396–402.
- Jensen, J.S. (2017). Mycoplasma genitalium: yet another challenging STI. The Lancet Infectious Diseases 17, 795–796.
- Joshi, S., Babu, J.M., Jayalakshmi, D., Kulkarni, V., Divate, U., Muwonge, R., Gheit, T., Tommasino, M., Sankaranarayanan, R., and Pillai, M.R. (2014). Human papillomavirus infection among human immunodeficiency virus-infected women in Maharashtra, India. Vaccine 32, 1079–1085.
- Jossens, R.M.O., Schachter, J., and Sweet, R.L. (1994). Risk Factors Associated With Pelvic Inflammatory Disease of Differing Microbial Etiologies. Obstetrics & Gynecology 83, 989.
- Lane, A.B., and Decker, C.F. (2016). *Chlamydia trachomatis* infections. Disease-a-Month 62, 269–273.
- Lee, J.E., Lee, S., Lee, H., Song, Y.-M., Lee, K., Han, M.J., Sung, J., and Ko, G. (2013). Association of the Vaginal Microbiota with Human Papillomavirus Infection in a Korean Twin Cohort. PLOS ONE 8, e63514.
- Ling, Z., Kong, J., Liu, F., Zhu, H., Chen, X., Wang, Y., Li, L., Nelson, K.E., Xia, Y., and Xiang, C. (2010). Molecular analysis of the diversity of vaginal microbiota associated with bacterial vaginosis. BMC Genomics 11, 488.

- Macklaim, J.M., Fernandes, A.D., Di Bella, J.M., Hammond, J.-A., Reid, G., and Gloor, G.B. (2013). Comparative meta-RNA-seq of the vaginal microbiota and differential expression by *Lactobacillus iners* in health and dysbiosis. Microbiome 1, 12.
- McDonald, A.C., Tergas, A.I., Kuhn, L., Denny, L., and Wright, T.C. (2014). Distribution of Human Papillomavirus Genotypes among HIV-Positive and HIV-Negative Women in Cape Town, South Africa. Front. Oncol. 4.
- Meltzer, M.C., Desmond, R.A., and Schwebke, J.R. (2008). Association of *Mobiluncus curtisii* With Recurrence of Bacterial Vaginosis. Sexually Transmitted Diseases 35, 611–613.
- Menon, S.S., Rossi, R., Harebottle, R., Mabeya, H., and vanden Broeck, D. (2016). Distribution of human papillomaviruses and bacterial vaginosis in HIV positive women with abnormal cytology in Mombasa, Kenya. Infect Agent Cancer 11.
- Moscicki, A.-B. (2005). Impact of HPV infection in adolescent populations. J Adolesc Health 37, S3-9.
- Murta, E.F.C., Souza, M.A.H. de, Araújo Júnior, E., and Adad, S.J. (2000). Incidence of *Gardnerella vaginalis*, *Candida* sp and human papilloma virus in cytological smears. Sao Paulo Medical Journal 118, 105–108.
- Ravel, J., Gajer, P., Abdo, Z., Schneider, G.M., Koenig, S.S.K., McCulle, S.L., Karlebach, S., Gorle, R., Russell, J., Tacket, C.O., et al. (2011). Vaginal microbiome of reproductive-age women. PNAS 108, 4680–4687.
- Schwebke, J.R., and Lawing, L.F. (2001). Prevalence of Mobiluncus spp Among Women With and Without Bacterial Vaginosis as Detected by Polymerase Chain Reaction. Sexually Transmitted Diseases 28, 195–199.
- Sellors, J.W., Mahony, J.B., Kaczorowski, J., Lytwyn, A., Bangura, H., Chong, S., Lorincz, A., Dalby, D.M., Janjusevic, V., and Keller, J.L. (2000). Prevalence and predictors of human papillomavirus infection in women in Ontario, Canada. CMAJ 163, 503–508.
- Shannon, B., Yi, T.J., Perusini, S., Gajer, P., Ma, B., Humphrys, M.S., Thomas-Pavanel, J., Chieza, L., Janakiram, P., Saunders, M., et al. (2017). Association of HPV infection and clearance with cervicovaginal immunology and the vaginal microbiota. Mucosal Immunol 10, 1310–1319.
- Soper, D.E., Brockwell, N.J., Dalton, H.P., and Johnson, D. (1994). Observations concerning the microbial etiology of acute salpingitis. American Journal of Obstetrics & Gynecology 170, 1008–1017.
- Srinivasan, S., Hoffman, N.G., Morgan, M.T., Matsen, F.A., Fiedler, T.L., Hall, R.W., Ross, F.J., McCoy, C.O., Bumgarner, R., Marrazzo, J.M., et al. (2012). Bacterial Communities in Women with Bacterial Vaginosis: High Resolution Phylogenetic Analyses Reveal Relationships of Microbiota to Clinical Criteria. PLOS ONE 7, e37818.
- Vidal, A.C., Murphy, S.K., Hernandez, B.Y., Vasquez, B., Bartlett, J.A., Oneko, O., Mlay, P., Obure, J., Overcash, F., Smith, J.S., et al. (2011). Distribution of HPV genotypes in cervical intraepithelial lesions and cervical cancer in Tanzanian women. Infect Agent Cancer 6, 20.
- Wu, X., Zhang, C., Zhu, X., and Wang, Y. (2010). Detection of HPV types and neutralizing antibodies in women with genital warts in Tianjin City, China. Virol. Sin. 25, 8–17.
- Xia, Q., Cheng, L., Zhang, H., Sun, S., Liu, F., Li, H., Yuan, J., Liu, Z., and Diao, Y. (2016). Identification of vaginal bacteria diversity and it's association with clinically diagnosed bacterial vaginosis by denaturing gradient gel electrophoresis and correspondence analysis. Infection, Genetics and Evolution 44, 479–486.
