# Supplementary material for: A novel sequencing-based vaginal health assay combining self-sampling, HPV detection and genotyping, STI detection, and vaginal microbiome analysis
Source: PLoS One. 2019 May 1;14(5):e0215945. doi: 10.1371/journal.pone.0215945 (PMC6493738; doi:10.1371/journal.pone.0215945)
Supplement: S2 Table — PPV, positive predictive value; NPV, negative predictive value. All values in the sensitivity, specificity, PPV, and NPV columns are given as percentages. Of the 72 selected targets, 31 passed selection criteria of all values above 90%. Values below 90% are shown in red. (PDF) [file pone.0215945.s004.pdf]

Supplementary material belonging to

"A novel sequencing-based vaginal health assay combining self-sampling, HPV detection and genotyping, STI detection, and vaginal microbiome analysis"

S2 Table. *In silico* performance metrics for the 72 bacterial targets (genus and species level) that were initially selected. PPV, positive predictive value; NPV, negative predictive value. All values in the sensitivity, specificity, PPV, and NPV columns are given as percentages. Of the 72 selected targets, 31 passed selection criteria of all values above 90%. Values below 90% are shown in red.

Target	Sensitivity	Specificity	PPV	NPV	Pass/Fail
Aerococcus	99.41	100.00	100.00	99.99	Pass
Atopobium	97.11	100.00	100.00	99.99	Pass
Fusobacterium	97.97	99.99	99.64	99.99	Pass
Gardnerella	97.91	100.00	100.00	99.99	Pass
Gemella	99.10	99.99	99.88	99.99	Pass
Lactobacillus	90.27	100.00	100.00	99.87	Pass
Megasphaera	97.96	99.99	99.84	99.99	Pass
Mobiluncus	100.00	100.00	100.00	100.00	Pass
Papillibacter	100.00	100.00	100.00	100.00	Pass
Parvimonas	98.35	99.99	99.81	99.99	Pass
Peptoniphilus	92.80	100.00	100.00	99.98	Pass
Peptostreptococcus	96.86	99.99	99.54	99.99	Pass

Porphyromonas	98.88	100.00	100.00	99.99	Pass
Prevotella	92.38	100.00	100.00	99.91	Pass
Sneathia	98.68	100.00	100.00	99.99	Pass
Acinetobacter baumannii	85.71	99.96	1.05	100.00	Fail
Aerococcus christensenii	100.00	100.00	100.00	100.00	Pass
Atopobium vaginae	100.00	100.00	100.00	100.00	Pass
Chlamydia trachomatis	98.23	100.00	100.00	100.00	Pass
Dialister micraerophilus	100.00	100.00	93.75	100.00	Pass
Fusobacterium nucleatum	97.06	100.00	92.03	100.00	Pass
Gardnerella vaginalis	100.00	100.00	100.00	100.00	Pass
Lactobacillus acidophilus	41.18	100.00	100.00	100.00	Fail
Lactobacillus alvei	100.00	100.00	16.67	100.00	Fail
Lactobacillus amylolyticus	100.00	100.00	83.33	100.00	Fail
Lactobacillus amylotrophicus	100.00	100.00	75.00	100.00	Fail
Lactobacillus animalis	100.00	100.00	48.84	100.00	Fail
Lactobacillus casei	95.60	99.98	68.15	100.00	Fail
Lactobacillus collinoides	100.00	100.00	14.29	100.00	Fail
Lactobacillus crispatus	93.75	100.00	51.37	100.00	Fail
Lactobacillus curvatus	30.36	100.00	100.00	100.00	Fail
Lactobacillus faecis	100.00	100.00	7.32	100.00	Fail
Lactobacillus fornicalis	100.00	100.00	6.67	100.00	Fail
Lactobacillus frumenti	25.00	100.00	100.00	100.00	Fail
Lactobacillus gasseri	97.73	99.99	49.71	100.00	Fail

Lactobacillus hilgardii	97.92	99.99	26.70	100.00	Fail
Lactobacillus hominis	100.00	99.99	1.27	100.00	Fail
Lactobacillus iners	95.00	100.00	100.00	100.00	Pass
Lactobacillus jensenii	100.00	100.00	94.74	100.00	Pass
Lactobacillus johnsonii	95.33	99.99	53.68	100.00	Fail
Lactobacillus koreensis	100.00	100.00	50.00	100.00	Fail
Lactobacillus leichmannii	100.00	99.99	0.90	100.00	Fail
Lactobacillus mali	77.78	100.00	87.50	100.00	Fail
Lactobacillus manihotivorans	50.00	100.00	100.00	100.00	Fail
Lactobacillus murinus	89.47	100.00	38.64	100.00	Fail
Lactobacillus nantensis	50.00	100.00	100.00	100.00	Fail
Lactobacillus oris	27.27	100.00	100.00	100.00	Fail
Lactobacillus otakiensis	100.00	99.99	3.45	100.00	Fail
Lactobacillus panis	25.00	100.00	100.00	100.00	Fail
Lactobacillus parabrevis	100.00	100.00	66.67	100.00	Fail
Lactobacillus parabuchneri	93.48	99.99	29.25	100.00	Fail
Lactobacillus pentosus	10.08	100.00	85.71	99.99	Fail
Lactobacillus reuteri	95.02	100.00	88.84	100.00	Fail
Lactobacillus sakei	98.05	100.00	76.26	100.00	Fail
Lactobacillus senioris	100.00	100.00	25.00	100.00	Fail
Lactobacillus similis	100.00	100.00	50.00	100.00	Fail
Lactobacillus sunkii	14.29	100.00	50.00	100.00	Fail
Lactobacillus taiwanensis	100.00	99.99	5.06	100.00	Fail

Lactobacillus vaginalis	76.67	100.00	100.00	100.00	Fail
Lactobacillus vini	100.00	100.00	83.33	100.00	Fail
Mobiluncus curtisii	100.00	100.00	100.00	100.00	Pass
Mobiluncus mulieris	100.00	100.00	100.00	100.00	Pass
Mycoplasma genitalium	100.00	100.00	100.00	100.00	Pass
Neisseria gonorrhoeae	100.00	100.00	97.92	100.00	Pass
Prevotella amnii	100.00	100.00	100.00	100.00	Pass
Prevotella timonensis	100.00	100.00	100.00	100.00	Pass
Staphylococcus aureus	99.80	99.94	93.60	100.00	Pass
Staphylococcus epidermidis	20.48	100.00	94.07	99.97	Fail
Streptococcus agalactiae	99.74	100.00	98.95	100.00	Pass
Ureaplasma parvum	100.00	100.00	31.82	100.00	Fail
Ureaplasma urealyticum	100.00	100.00	75.29	100.00	Fail
Veillonella parvula	83.33	100.00	58.82	100.00	Fail
