# Supplementary material for: A novel sequencing-based vaginal health assay combining self-sampling, HPV detection and genotyping, STI detection, and vaginal microbiome analysis
Source: PLoS One. 2019 May 1;14(5):e0215945. doi: 10.1371/journal.pone.0215945 (PMC6493738; doi:10.1371/journal.pone.0215945)
Supplement: S3 Table — HPV, human papillomavirus; TP, true positive; FN, false negative; FP, false positive; TN, true negative; Sens, sensitivity (in %); Spec, specificity (in %); PPV, positive predictive value (in %); NPV, negative predictive value (in %). (PDF) [file pone.0215945.s005.pdf]

Supplementary material belonging to

"A novel sequencing-based vaginal health assay combining self-sampling, HPV detection and genotyping, STI detection, and vaginal microbiome analysis"

S3 Table. *In silico* performance metrics for the 19 HPV targets. HPV, human papillomavirus; TP, true positive; FN, false negative; FP, false positive; TN, true negative; Sens, sensitivity (in %); Spec, specificity (in %); PPV, positive predictive value (in %); NPV, negative predictive value (in %).

Type	Risk type	TP	FN	FP	TN	Sens	Spec	PPV	NPV	Status
HPV6	Low-risk	13438	188	0	147772	98.62	100.00	100.00	99.87	Pass
HPV11	Low-risk	9900	182	0	151316	98.19	100.00	100.00	99.88	Pass
HPV16	High-risk	53378	1470	0	106550	97.32	100.00	100.00	98.64	Pass
HPV18	High-risk	7146	10	0	154242	99.86	100.00	100.00	99.99	Pass
HPV31	High-risk	3620	72	0	157706	98.05	100.00	100.00	99.95	Pass
HPV33	High-risk	2178	0	0	159220	100.00	100.00	100.00	100.00	Pass
HPV35	High-risk	2595	0	0	158803	100.00	100.00	100.00	100.00	Pass
HPV39	High-risk	1030	0	0	160368	100.00	100.00	100.00	100.00	Pass
HPV42	Low-risk	2080	0	0	159318	100.00	100.00	100.00	100.00	Pass
HPV43	Low-risk	110	0	0	161288	100.00	100.00	100.00	100.00	Pass
HPV44	Low-risk	320	20	0	161058	94.12	100.00	100.00	99.99	Pass
HPV45	High-risk	1200	0	0	160198	100.00	100.00	100.00	100.00	Pass
HPV51	High-risk	1280	20	0	160098	98.46	100.00	100.00	99.99	Pass
HPV52	High-risk	19255	0	0	142143	100.00	100.00	100.00	100.00	Pass

HPV56	High-risk	560	20	0	160818	96.55	100.00	100.00	99.99	Pass
HPV58	High-risk	27283	659	0	133456	97.64	100.00	100.00	99.51	Pass
HPV59	High-risk	154	0	0	161244	100.00	100.00	100.00	100.00	Pass
HPV66	High-risk	615	0	0	160783	100.00	100.00	100.00	100.00	Pass
HPV68	High-risk	1220	0	0	160178	100.00	100.00	100.00	100.00	Pass
