# Supplementary material for: A novel sequencing-based vaginal health assay combining self-sampling, HPV detection and genotyping, STI detection, and vaginal microbiome analysis
Source: PLoS One. 2019 May 1;14(5):e0215945. doi: 10.1371/journal.pone.0215945 (PMC6493738; doi:10.1371/journal.pone.0215945)
Supplement: S4 Table — Sequences are based on the 16S rRNA gene. (PDF) [file pone.0215945.s006.pdf]

Supplementary material belonging to

"A novel sequencing-based vaginal health assay combining self-sampling, HPV detection and genotyping, STI detection, and vaginal microbiome analysis"

S4 Table. List of the 31 synthetic DNAs created to represent the bacterial targets included in the assay. Sequences are based on the 16S rRNA gene.

Microorganism	Sequence
Aerococcus	TATAAGAGAAGAACAAATTGTAGAGTAACTGCTACAGTCTTGACGGTATCTTATCAGAAAGCC<br>ACGGCTAACTACGTGCCAGCAGCCGCGGTAATACGTAGGTGGCAAGCGTTGTCCGGATTTATT<br>GGGCGTAAAGGGAGCGCAGGTGGTTTCTTAAGTCTGATGTGAAAGCCCACGGCTTAACCGTGG<br>AGGGTCATTGGAAACTGGGAAACTTGAGTACAGAAGAGGAATGTGGAACTCCATGTGTAGCGG<br>TGGAATGCGTAGATATATGGAAGAACACCAGTGGCGAAGGCGACATTCTGGTCTGTTACTGAC<br>ACTGAGGCTCGAAAGCGTGGGGAGCAAACAGGATTAGATACCCTGGTAGTCCACGCCGTAAAC<br>GATGAGTGCTAGGTGTTGGAGGGTTTCCGCCCTTCAGTGCCGCAGTTAACGCATTAAGCACTC<br>C
Aerococcus christensenii	TGTAAGAGAAGAACAAATTGTAGAGTAACTGCTACAGTCTTGACGGTATCTTACCAGAAAGCC<br>ACGGCTAACTACGTGCCAGCAGCCGCGGTAATACGTAGGTGGCAAGCGTTGTCCGGATTTATT<br>GGGCGTAAAGGGGGCGCAGGCTGCTTCTTAAGTCTGATGTGAAAGCCCACGGCTTAACCGTGG<br>AAGTGCATTGGAAACTGGGAAGCTTGAGTACAGAAGAGGAAAGTGGAACTCCATGTGTAGCGG<br>TGGAATGCGTAGATATATGGAAGAACACCAGTGGCGAAAGCGACTTTCTGGTCTGTCACTGAC<br>GCTGAGGCCCGAAAGCGTGGGTAGCAAACAGGATTAGATACCCTGGTAGTCCACGCCGTAAAC<br>GATGAGCGCTAGGTGTTGGAGGGTTTCCACCCTTCAGTGCCGCAGCTAACGCATTAAGCGCTC<br>C
Atopobium	TTCGGGTTGTAAACCGCTTTCAGCAGGGACGAGGCGAAAGTGACGGTACCTGCAGAAGAAGCC

	CCGGCTAACTACGTGCCAGCAGCCGCGGTAATACGTAGGGGGCAAGCGTTATCCGGATTCATT<br>GGGCGTAAAGCGCTCGTAGGCGGTCTGTTAGGTCGGGAGTTAAATCCGGGGGCTCAACCCCCG<br>CTCGCTCCCGATACCGGCAGACTTGAGTTTGGTAGGGGAAGGTGGAATTCCTAGTGTAGCGGT<br>GGAATGCGCAGATATTAGGAAGAACACCAGTGGCGAAGGCGGCCTTCTGGGCCATAACTGACG<br>CTGAGGAGCGAAAGCTAGGGGAGCAAACAGGATTAGATACCCTGGTAGTCCTAGCCGTAAACG<br>ATGGACACTAGGTGTGGGGGAATATTTCTTCCGTGCCGCAGCTAACGCATTAAGTGTCCCGCC
Atopobium vaginae	CGGGTTGTAAACCGCTTTCAGCAGGGACCAGGCCGCAAGGTGACGGTACCTGCACAAGAAGCC<br>CCGGCTAACTACGTGCCAGCAGCCGCGGTAATACGTAGGGGGCAAGCGTTATCCGGATTCATT<br>GGGCGTAAAGCGCGCGTAGGCGGTCTGTTAGGTCAGGAGTTAAATCTGGGGGCTCAACCCCTA<br>TCCGCTCCTGATACCGGCAGGCTTGAGTCTGGTAGGGGAAGATGGAATTCCAAGTGTAGCGGT<br>GAAATGCGCAGATATTTGGAAGAACACCGGTGGCGAAGGCGGTCTTCTGGGCCATGACTGACG<br>CTGAGGCGCGAAAGCTAGGGGAGCGAACAGGATTAGATACCCTGGTAGTCCTACCTGTAAACG<br>ATGGACACTAGGTGTGGGGAGATTATACTTTCCGTGCCGCAGCTAACGCATTAAGTGTCCCGC
Chlamydia trachomatis	CGCTTGGGAATAAGAGAAGGCGGTTAATACCCGCTGGATTTGAGCGTACCAGGTAAAGAAGCA<br>CCGGCTAACTCCGTGCCAGCAGCTGCGGTAATACGGAGGGTGCTAGCGTTAATCGGATTTATT<br>GGGCGTAAAGGGCGTGTAGGCGGAAAGGTAAGTTAGTTGTCAAAGATCGGGGCTCAACCCCGA<br>GTCGGCATCTAATACTATTTTTCTAGAGGGTAGATGGAGAAAAGGGAATTTCACGTGTAGCGG<br>TGAAATGCGTAGATATGTGGAAGAACACCAGTGGCGAAGGCGCTTTTCTAATTTATACCTGAC<br>GCTAAGGCGCGAAAGCAAGGGGAGCAAACAGGATTAGATACCCTGGTAGTCCTTGCCGTAAAC<br>GATGCATACTTGATGTGGATGGTCTCAACCCCATCCGTGTCGGAGCTAACGCGTTAAGTATGC<br>C
Dialister micraerophilus	GATTCGGGACGAAAGGCCATATGTGAATAATATATGGAAATGACGGTACCGAAAAAGCAAGCC<br>ACGGCTAACTACGTGCCAGCAGCCGCGGTAATACGTAGGTGGCAAGCGTTGTCCGGAATTATT<br>GGGCGTAAAGCGCGCGCAGGCGGTCACTTAAGTCCATCTTAGAAGTGCGGGGCTTAACCCCGT<br>GATGGGATGGAAACTGGGAGACTGGAGTATCGGAGAGGAAAGTGGAATTCCTAGTGTAGCGGT<br>GAAATGCGTAGATATTAGGAAGAACACCGGTGGCGAAGGCGACTTTCTGGACGAAAACTGACG<br>CTGAGGCGCGAAAGCGTGGGGAGCAAACAGGATTAGATACCCTGGTAGTCCACGCCGTAAACG

	ATGGATACTAGGTGTAGGAGGTATCGACCCCTTCTGTGCCGGAGTTAACGCAATAAGTATCCC
Fusobacterium	TTTTCGGAATGTAAAGTGCTTTCAGTTGGGAAGAAAGAAATGACGGTACCAACAGAAGAAGTG ACGGCTAAATACGTGCCAGCAGCCGCGGTAATACGTATGTCACGAGCGTTATCCGGATTTATT GGGCGTAAAGCGCGTCTAGGTGGTTATGTAAGTCTGATGTGAAAATGCAGGGCTCAACTCTGT ATTGCGTTGGAAACTGTATAACTAGAGTACTGGAGAGGTAAGCGGAACTACAAGTGTAGAGGT GAAATTCGTAGATATTTGTAGGAATGCCGATGGGGAAGCCAGCTTACTGGACAGATACTGACG CTAAAGCGCGAAAGCGTGGGTAGCAAACAGGATTAGATACCCTGGTAGTCCACGCTGTAAACG ATGATTACTAGGTGTTGGGGGTCGAACCTCAGCGCCCAAGCAAACGCGATAAGTAATCCGCCT
Fusobacterium nucleatum	TTTTCGGAATGTAAAGTGCTTTCAGTTGGGAAGAAAAAAATGACGGTACCAACAGAAGAAGTG ACGGCTAAATACGTGCCAGCAGCCGCGGTAATACGTATGTCACGAGCGTTATCCGGATTTATT GGGCGTAAAGCGCGTCTAGGTGGTTATATAAGTCTGATGTGAAAATGCAGGGCTCAACTCTGT ATTGCGTTGGAAACTGTATAACTAGAGTACTGGAGAGGTAAGCGGAACTACAAGTGTAGAGGT GAAATTCGTAGATATTTGTAGGAATGCCGATGGGGAAGCCAGCTTACTGGACAGATACTGACG CTGAAGCGCGAAAGCGTGGGTAGCAAACAGGATTAGATACCCTGGTAGTCCACGCCGTAAACG ATGATTACTAGGTGTTGGGGGTCGAACCTCAGCGCCCAAGCAAACGCGATAAGTAATCCGCCT
Gardnerella	GGGTTGTAAACCGCTTTTGATTGGGAGCAAGCCTTTTGGGTGAGTGTACCTTTCGAATAAGCG CCGGCTAACTACGTGCCAGCAGCCGCGGTAATACGTAGGGCGCAAGCGTTATCCGGAATTATT GGGCGTAAAGAGCTTGTAGGCGGTTCGTCGCGTCTGGTGTGAAAGCCCATCGCTTAACGGTGG GTTTGCGCCGGGTACGGGCGGGCTAGAGTGCAGTAGGGGAGACTGAAATTCTCGGTGTAACGG TGGAATGTGTAGATATCGGGAAGAACACCAATGGCGAAGGCAGGTCTCTGGGCTGTTACTGAC GCTGAGAAGCGAAAGCGTGGGGAGCGAACAGGATTAGATACCCTGGTAGTCCACGCCGTAAAC GGTGGACGCTGGATGTGGGGCCCATTCCACGGGTTCTGTGTCGGAGCTAACGCGTTAAGCGTC C
Gardnerella vaginalis	CGGGTTGTAAACCGCTTTTGATTGGGAGCAAGCTTTCGGGTGAGTGTACCTTTCGAATAAGCG CCGGCTAACTACGTGCCAGCAGCCGCGGTAATACGTAGGGCGCAAGCGTTATCCGGAATTATT GGGCGTAAAGAGCTTGTAGGCGGTTCGTCGCGTCTGGTGTGAAAGCCCATCGCTTAACGGTGG GTTTGCGCCGGGTACGGGCGGGCTAGAGTGCAGTAGGGGAGACTGGAATTCCCGGTGTAACGG

	TGGAATGTGTAGATATCGGGAAGAACACCAATGGCGAAGGCAGGTCTCTGGGCTGTTACTGAC<br>GCTGAGAAGCGAAAGCGTGGGGAGCGAACAGGATTAGATACCCTGGTAGTCCACGCCGTAAAC<br>GGTGGACGCTGGATGTGGGGCCCATTCCACGGGTTCCGTGTCGGAGCTAACGCGTTAAGCGTC<br>C
Gemella	TGTTAGGGAAGAATGATTGTGTAGTAACTATACACAGTAGAGACGGTACCTAACCAGAAAGCC<br>ACGGCTAACTACGTGCCAGCAGCCGCGGTAATACGTAGGTGGCAAGCGTTGTCCGGAATTATT<br>GGGCGTAAAGCGCGCGCAGGTGGTTTAATAAGTCTGATGTGAAAGCCCACGGCTCAACCGTGG<br>AGGGTCATTGGAAACTGTTAAACTTGAGTGCAGGAGAGAAAAGTGGAATTCCTAGTGTAGCGG<br>TGAAATGCGTAGAGATTAGGAGGAACACCAGTGGCGAAGGCGGCTTTTTGGCCTGTAACTGAC<br>ACTGAGGCGCGAAAGCGTGGGGAGCAAACAGGATTAGATACCCTGGTAGTCCACGCCGTAAAC<br>GATGAGTGCTAAGTGTTGGTCTCATAAGAGATCAGTGCTGCAGCTAACGCATTAAGCACTCCG<br>C
Lactobacillus	TGGTAGTGAAGAAAGATAGAGGTAGTAACTGGCCTTTATTTGACGGTAATTACTTAGAAAGTC<br>ACGGCTAACTACGTGCCAGCAGCCGCGGTAATACGTAGGTGGCAAGCGTTGTCCGGATTTATT<br>GGGCGTAAAGCGAGTGCAGGCGGTTCAATAAGTCTGATGTGAAAGCCTTCGGCTCAACCGGAG<br>AATTGCATCAGAAACTGTTGAACTTGAGTGCAGAAGAGGAGAGTGGAACTCCATGTGTAGCGG<br>TGGAATGCGTAGATATATGGAAGAACACCAGTGGCGAAGGCGGCTCTCTGGTCTGCAACTGAC<br>GCTGAGGCTCGAAAGCATGGGTAGCGAACAGGATTAGATACCCTGGTAGTCCATGCCGTAAAC<br>GATGAGTGCTAAGTGTTGGGAGGTTTCCGCCTCTCAGTGCTGCAGCTAACGCATTAAGCACTC<br>C
Lactobacillus iners	TGTTGGTGAAGAAGGACAGGGGTAGTAACTGACCTTTGTTTGACGGTAATCAATTAGAAAGTC<br>ACGGCTAACTACGTGCCAGCAGCCGCGGTAATACGTAGGTGGCAAGCGTTGTCCGGATTTATT<br>GGGCGTAAAGCGAGTGCAGGCGGCTCGATAAGTCTGATGTGAAAGCCTTCGGCTCAACCGGAG<br>AATTGCATCAGAAACTGTCGAGCTTGAGTACAGAAGAGGAGAGTGGAACTCCATGTGTAGCGG<br>TGAAATGCGTAGATATATGGAAGAACACCGGTGGCGAAGGCGGCTCTCTGGTCTGTTACTGAC<br>GCTGAGGCTCGAAAGCATGGGTAGCGAACAGGATTAGATACCCTGGTAGTCCATGCCGTAAAC<br>GATGAGTGCTAAGTGTTGGGAGGTTTCCGCCTCTCAGTGCTGCAGCTAACGCATTAAGCACTC

	C
Lactobacillus jensenii	TGTTGGTGAAGAAGGATAGAGGTAGTAACTGGCCTTTATTTGACGGTAATCAACCAGAAAGTCACGGCTAACTACGTGCCAGCAGCCGCGGTAATACGTAGGTGGCAAGCGTTGTCCGGATTTATTGGGCGTAAAGCGAGCGCAGGCGGATTGATAAGTCTGATGTGAAAGCCTTCGGCTCAACCGAAGAACTGCATCAGAAACTGTCAATCTTGAGTGCAGAAGAGGAGAGTGGAACTCCATGTGTAGCGGTGGAATGCGTAGATATATGGAAGAACACCAGTGGCGAAGGCGGCTCTCTGGTCTGTAACTGACGCTGAGGCTCGAAAGCATGGGTAGCGAACAGGATTAGATACCCTGGTAGTCCATGCCGTAAACGATGAGTGCTAAGTGTTGGGAGGTTTCCGCCTCTCAGTGCTGCAGCTAACGCATTAAGCACTCC
Megasphaera	ATATGGGACGAACAGGACATCGGTTAATACCCGGTGTCTTTGACGGTACCGTAAGAGAAAGCCACGGCTAACTACGTGCCAGCAGCCGCGGTAATACGTAGGTGGCAAGCGTTGTCCGGAATTATTGGGCGTAAAGGGCGCGCAGGCGGCATCGCAAGTCGGTCTTAAAAGTGCGGGGCTTAACCCCGTGAGGGGACCGAAACTGTGAAGCTCGAGTGTCGGAGAGGAAAGCGGAATTCCTAGTGTAGCGGTGAAATGCGTAGATATTAGGAGGAACACCAGTGGCGAAAGCGGCTTTCTGGACGACAACTGACGCTGAGGCGCGAAAGCCAGGGGAGCAAACGGGATTAGATACCCCGGTAGTCCTGGCCGTAAACGATGGATACTAGGTGTAGGAGGTATCGACTCCTTCTGTGCCGGAGTTAACGCAATAAGTATCCC
Mobiluncus	ACTCCTTTTTCTCGCGAAAAAGGCACAGCTTTGGCTGTGTTGATGGTAGTGGGGGAAGAAGCGCCGGCTAACTACGTGCCAGCAGCCGCGGTAATACGTAGGGCGCGAGCGTTGTCCGGATTTATTGGGCGTAAAGAGCTCGTAGGTGGTTCGTCGCGTCTGTCGTGAAAGCCAGCAGCTTAACTGTTGGTCTGCGGTGGGTACGGGCGGGCTTGAGTGCGGTATGGGTGACTGGAATTCCTGGTGTAGCGGTGGAATGCGCAGATATCAGGAGGAACACCGATGGCGAAGGCAGGTCACTGGGCCGTTACTGACACTGAGGAGCGAAAGCGTGGGGAGCGAACAGGATTAGATACCCTGGTAGTCCACGCTGTAAACGTTGGGAACTAGGTGTGGGGATGCTATCCTGTGTTTCTGCGCCGTAGCTAACGCATTAAGTTCC
Mobiluncus curtisii	ACTCCTTTTTCTCGCGAAAAAGGCACAGTTTTGGCTGTGTTGATGGTAGTGGGGGAAGAAGCGCCGGCTAACTACGTGCCAGCAGCCGCGGTAATACGTAGGGCGCGAGCGTTGTCCGGATTTATTGGGCGTAAAGAGCTCGTAGGTGGTTCGTCGCGTCTGTCGTGAAAGCCAGCAGCTTAACTGTTG

	GTCTGCGGTGGGTACGGGCGGGCTTGAGTGCGGTAGGGGTGACTGGAATTCCTGGTGTAGCGG<br>TGGAATGCGCAGATATCAGGAGGAACACCGATGGCGAAGGCAGGTCACTGGGCCGTTACTGAC<br>ACTGAGGAGCGAAAGCGTGGGGAGCGAACAGGATTAGATACCCTGGTAGTCCACGCTGTAAAC<br>GTTGGGAACTAGGTGTGGGGATGCTATCCTGTGTCTCTGCGCCGTAGCTAACGCATTAAGTTC<br>C
Mobiluncus mulieris	ACTCCTTTTTCTCGTGAAAAAGGCATGCTTTTGGGTGTGTTGATGGTAGCGGGGGAAGAAGCG<br>CCGGCTAACTACGTGCCAGCAGCCGCGGTAATACGTAGGGCGCGAGCGTTGTCCGGATTTATT<br>GGGCGTAAAGAGCTCGTAGGTGGTTCGTCGCGTCTGTCGTGAAAGCCAGCAGCTTAACTGTTG<br>GTCTGCGGTGGGTACGGGCGGGCTTGAGTGCGGTAGGGGTGACTGGAATTCCTGGTGTAGCGG<br>TGGAATGCGCAGATATCAGGAGGAACACCGATGGCGAAGGCAGGTCACTGGGCCGTTACTGAC<br>GCTGAGGAGCGAAAGCGTGGGGAGCGAACAGGATTAGATACCCTGGTAGTCCACGCTGTAAAC<br>GTTGGGAACTAGGTGTGGGGATGCTATCCTGTGTTTCTGCGCCGTAGCTAACGCATTAAGTTC<br>C
Mycoplasma genitalium	ATTTGGGAAGAATGACTCTAGCAGGCAATGGCTGGAGTTTGACTGTACCACTTTGAATAAGTG<br>ACGACTAACTATGTGCCAGCAGTCGCGGTAATACATAGGTCGCAAGCGTTATCCGGATTTATT<br>GGGCGTAAAGCAAGCGCAGGCGGATTGAAAAGTCTGGTGTTAAAGGCAGCTGCTTAACAGTTG<br>TATGCATTGGAAACTATCAGTCTAGAGTGTGGTAGGGAGTTTTGGAATTTCATGTGGAGCGGT<br>GAAATGCGTAGATATATGAAGGAACACCAGTGGCGAAGGCGAAAACTTAGGCCATTACTGACG<br>CTTAGGCTTGAAAGTGTGGGGAGCAAATAGGATTAGATACCCTAGTAGTCCACACCGTAAACG<br>ATAGATACTAGCTGTCGGAGCGATCCCTTCGGTAGTGAAGTTAACACATTAAGTATCTCGCCT
Neisseria gonorrhoeae	TGTCAGGGAAGAAAAGGCTGTTGCCAATATCGGCGGCCGATGACGGTACCTGAAGAATAAGCA<br>CCGGCTAACTACGTGCCAGCAGCCGCGGTAATACGTAGGGTGCGAGCGTTAATCGGAATTACT<br>GGGCGTAAAGCGGGCGCAGACGGTTACTTAAGCAGGATGTGAAATCCCCGGGCTCAACCCGGG<br>AACTGCGTTCTGAACTGGGTGACTCGAGTGTGTCAGAGGGAGGTGGAATTCCACGTGTAGCAG<br>TGAAATGCGTAGAGATGTGGAGGAATACCGATGGCGAAGGCAGCCTCCTGGGATAACACTGAC<br>GTTCATGTCCGAAAGCGTGGGTAGCAAACAGGATTAGATACCCTGGTAGTCCACGCCCTAAAC<br>GATGTCAATTAGCTGTTGGGCAACTTGATTGCTTGGTAGCGTAGCTAACGCGTGAAATTGACC

	G
Papillibacter	AGGCTTTCGGGTTGTAAACTCCTTTGACGAGGGACGATGATGACGGTACCTCGAAAACAAGCCACGGCTAACTACGTGCCAGCAGCCGCGGTAATACGTAGGTGGCAAGCGTTGTCCGGATTTACTGGGTGTAAAGGGCGCGTAGGCGGGCAGGCAAGTCAGATGTGAAATCTCCGGGCTCAACCCGGAAATTGCATTTGAAACTGCAGGTCTTGAGTATCGGAGAGGCAAGCGGAATTCCTAGTGTAGCGGTGAAATGCGTAGATATTAGGAGGAACACCAGTGGCGAAGGCGGCTTGCTGGACGACAACTGACGCTGAGGCGCGAAAGCGTGGGGAGCAAACAGGATTAGATACCCTGGTAGTCCATGCCCTAAACGATGAATATATGTGTGGGGGGACTGACCCCTTCCGTGCCGGAGTAACACAATAAGTATTCCACC
Parvimonas	AGGTTTTCGAATCGTAAAGCTCTGTCCTATGAGAAGATAATGACGGTATCATAGGAGGAAGCCCCGGCTAAATACGTGCCAGCAGCCGCGGTAATACGTATGGGGCGAGCGTTGTCCGGAATTATTGGGCGTAAAGGGTACGTAGGCGGTTTTTTAAGTCAGGTGTGAAAGCGTGAGGCTTAACCTCATTAAGCACTTGAAACTGGAAGACTTGAGTGAAGGAGAGGAAAGTGGAATTCCTAGTGTAGCGGTGAAATGCGTAGATATTAGGAGGAATACCGGTGGCGAAGGCGACTTTCTGGACTTTTACTGACGCTCAGGTACGAAAGCGTGGGGAGCAAACAGGATTAGATACCCTGGTAGTCCACGCCGTAAACGATGAATGCTAGGTGTTGGGAGTCAAATCTCGGTGCCGAAGTTAACACATTAAGCATTCCGCCT
Peptoniphilus	AGGCTTTCGAGTCGTAAAGTTCTTTTATATGGGAAGATAATGACGGTACCATAAGAAAAAGCCCCGGCTAACTACGTGCCAGCAGCCGCGGTAATACGTAGGGGGCTAGCGTTGTCCGGAATCACTGGGCGTAAAGGGTTCGCAGGCGGAAATGCAAGTCAGATGTAAAAGGCAGTAGCTTAACTACTGTAAGCATTTGAAACTGCATATCTTGAGAAGAGTAGAGGTAAGTGGAATTTTTAGTGTAGCGGTGAAATGCGTAGATATTAAAAAGAATACCGGTGGCGAAGGCGACTTACTGGGCTCATTCTGACGCTGAGGAACGAAAGCGTGGGTAGCAAACAGGATTAGATACCCTGGTAGTCCACGCTGTAAACGATGAGTGCTAGGTATCGGAATAATTCGGTGCCGCAGTTAACACATTAAGCACTCCGCCTGGGG
Peptostreptococcus	AGGTCTTCGGATCGTAAAGTTCTGTTGCAGGGGAAGATAATGACGGTACCCTGTGAGGAAGCCCCGGCTAACTACGTGCCAGCAGCCGCGGTAATACGTAGGGGGCTAGCGTTATCCGGATTTACTGGGCGTAAAGGGTGCGTAGGTGGTCCTTCAAGTCGGTGGTTAAAGGCTACGGCTCAACCGTAGTAAGCCGCCGAAACTGGAGGACTTGAGTGCAGGAGAGGAAAGTGGAATTCCCAGTGTAGCGGT

	GAAATGCGTAGATATTGGGAGGAACACCAGTAGCGAAGGCGGCTTTCTGGACTGCAACTGACA<br>CTGAGGCACGAAAGCGTGGGTAGCAAACAGGATTAGATACCCTGGTAGTCCACGCTGTAAACG<br>ATGAGTACTAGGTGTCGGGGGTTACCCCCCTCGGTGCCGCAGCTAACGCATTAAGTACTCCGC
Porphyromonas	TTCTTTTGTAGGGGAATAACGGACGGCACGTGTGCCGTAGTGAATGTACCCTACGAATAAGCA<br>TCGGCTAACTCCGTGCCAGCAGCCGCGGTAATACGGAGGATGCGAGCGTTATCCGGATTTATT<br>GGGTTTAAAGGGTGCGTAGGCGGCCTGTTAAGTCAGCGGTGAAATCTAGGAGCTTAACTCCTA<br>AATTGCCATTGATACTGGCGGGCTTGAGTGTAGATGAGGTAGGCGGAATGCGTGGTGTAGCGG<br>TGGAATGCATAGATATCACGCAGAACTCCAATTGCGAAGGCAGCTTACTAAGGTACAACTGAC<br>GCTGAAGCACGAAAGCGTGGGTATCAAACAGGATTAGATACCCTGGTAGTCCACGCAGTAAAC<br>GATGATAACTGGGCGTATGCGATATACAGTATGCTCCTAAGCGAAAGCGTTAAGTTATCCACC<br>T
Prevotella	TGCTTTTATAAGGGAATAAAGTGAGTCTCGTGAGACTTTTTGCATGTACCTTATGAATAAGGA<br>CCGGCTAATTCCGTGCCAGCAGCCGCGGTAATACGGAAGGTCCGGGCGTTATCCGGATTTATT<br>GGGTTTAAAGGGAGCGTAGGCCGGAGATTAAGCGTGTTGTGAAATGTAGAAGCTCAACGTCTG<br>CACTGCAGCGCGAACTGGTTTCCTTGAGTACGTACAAAGTGGGCGGAATTCGTGGTGTAGCGG<br>TGAAATGCTTAGATATCACGAAGAACTCCGATTGCGAAGGCAGCTCACTGGAGCGCAACTGAC<br>GCTGAAGCTCGAAAGTGCGGGTATCGAACAGGATTAGATACCCTGGTAGTCCGCACGGTAAAC<br>GATGGATGCCCGCTGTTGGTCTGAATAGGTCAGCGGCCAAGCGAAAGCATTAAGCATCCCACC<br>T
Prevotella amnii	TGCTTTTATATGGGAATAAAGTGAGGGACGTGTCCCTTATTGCATGTACCATATGAATAAGGA<br>CCGGCTAATTCCGTGCCAGCAGCCGCGGTAATACGGAAGGTCCAGGCGTTATCCGGATTTATT<br>GGGTTTAAAGGGAGCGTAGGCTGTTTGTTAAGCGTGTTGTGAAATGTAGGAGCTCAACTTTTA<br>GATTGCAGCGCGAACTGGCAGACTTGAGTGCGCACAACGTAGGCGGAATTCATGGTGTAGCGG<br>TGAAATGCTTAGATATCATGACGAACTCCGATTGCGAAGGCAGCTTACGGGAGCGCAACTGAC<br>GCTAAAGCTCGAAGGTGCGGGTATCGAACAGGATTAGATACCCTGGTAGTCCGCACAGTAAAC<br>GATGGATGCCCGCTGTTAGCACCTAGTGTTAGCGGCTAAGCGAAAGCATTAAGCATCCCACCT<br>G

Prevotella timonensis	TGCTTTTATGTGGGGATAAAGTGCGTGACGTGTCATGCATTGCAGGTACCACATGAATAAGGA<br>CCGGCTAATTCCGTGCCAGCAGCCGCGGTAATACGGAAGGTCCGGGCGTTATCCGGATTTATT<br>GGGTTTAAAGGGAGCGTAGGCTGTCTATTAAGCGTGTTGTGAAATTTACCGGCTCAACCGGTG<br>GCTTGCAGCGCGAACTGGTCGACTTGAGTATGCAGGAAGTAGGCGGAATTCATGGTGTAGCGG<br>TGAAATGCTTAGATATCATGACGAACTCCGATTGCGCAGGCAGCTTACTGTAGCATAACTGAC<br>GCTGATGCTCGAAAGTGCGGGTATCAAACAGGATTAGATACCCTGGTAGTCCGCACGGTAAAC<br>GATGGATGCTCGCTATTCGTCCTTTTTGGATGAGTGGCCAAGTGAAAACATTAAGCATCCCAC<br>C
Sneathia	GTTTTAGGACTGTAAAACACTTTTAGTAGGGAAGAAAAAATGACGGTACCTACAGAAGAAGCG<br>ACGGCTAAATACGTGCCAGCAGCCGCGGTAATACGTATGTCGCGAGCGTTATCCGGAATTATT<br>GGGCTTAAAGGGCATCTAGGCGGTTAAACAAGTTGAAGGTGAAAACCTGTGGCTCAACCATAG<br>GCTTGCCTACAAAACTGTATAACTAGAGTACTGGAAAGGTGGGTGGAACTACACGAGTAGAGG<br>TGAAATTCGTAGATATGTGTAGGAATGCCGATGATGAAGATAACTCACTGGACAGCAACTGAC<br>GCTGAAGTGCGAAAGCTAGGGGAGCAAACAGGATTAGATACCCTGGTAGTCCTAGCTGTAAAC<br>GATGATCACTGGGTGTGGGGATTCGAAGTCTCTGTGCCGAAGCAAAAGCGATAAGTGATCCGC<br>C
Staphylococcus aureus	TATTAGGGAAGAACATATGTGTAAGTAACTGTGCACATCTTGACGGTACCTAATCAGAAAGCC<br>ACGGCTAACTACGTGCCAGCAGCCGCGGTAATACGTAGGTGGCAAGCGTTATCCGGAATTATT<br>GGGCGTAAAGCGCGCGTAGGCGGTTTTTTAAGTCTGATGTGAAAGCCCACGGCTCAACCGTGG<br>AGGGTCATTGGAAACTGGAAAACTTGAGTGCAGAAGAGGAAAGTGGAATTCCATGTGTAGCGG<br>TGAAATGCGCAGAGATATGGAGGAACACCAGTGGCGAAGGCGACTTTCTGGTCTGTAACTGAC<br>GCTGATGTGCGAAAGCGTGGGGATCAAACAGGATTAGATACCCTGGTAGTCCACGCCGTAAAC<br>GATGAGTGCTAAGTGTTAGGGGGTTTCCGCCCCTTAGTGCTGCAGCTAACGCATTAAGCACTC<br>C
Streptococcus agalactiae	GTTAGAGAAGAACGTTGGTAGGAGTGGAAAATCTACCAAGTGACGGTAACTAACCAGAAAGGG<br>ACGGCTAACTACGTGCCAGCAGCCGCGGTAATACGTAGGTCCCGAGCGTTGTCCGGATTTATT<br>GGGCGTAAAGCGAGCGCAGGCGGTTCTTTAAGTCTGAAGTTAAAGGCAGTGGCTTAACCATTG

	TACGCTTTGGAAACTGGAGGACTTGAGTGCAGAAGGGGAGAGTGGAATTCCATGTGTAGCGGT<br><br>GAAATGCGTAGATATATGGAGGAACACCGGTGGCGAAAGCGGCTCTCTGGTCTGTAACTGACG<br><br>CTGAGGCTCGAAAGCGTGGGGAGCAAACAGGATTAGATACCCTGGTAGTCCACGCCGTAAACG<br><br>ATGAGTGCTAGGTGTTAGGCCCTTTCCGGGGCTTAGTGCCGCAGCTAACGCATTAAGCACTCC
