# Supplementary material for: A novel sequencing-based vaginal health assay combining self-sampling, HPV detection and genotyping, STI detection, and vaginal microbiome analysis
Source: PLoS One. 2019 May 1;14(5):e0215945. doi: 10.1371/journal.pone.0215945 (PMC6493738; doi:10.1371/journal.pone.0215945)
Supplement: S5 Table — Because of sequence variability, hrHPV type 68 was represented by 2 different sDNAs. (PDF) [file pone.0215945.s007.pdf]

Supplementary material belonging to

"A novel sequencing-based vaginal health assay combining self-sampling, HPV detection and genotyping, STI detection, and vaginal microbiome analysis"

S5 Table. List of the 20 synthetic DNA sequences representing 5 lrHPV and 14 hrHPV types included in the assay. Because of sequence variability, hrHPV type 68 was represented by 2 different sDNAs.

HPV type	Risk type	Sequence
HPV6	Low-risk	TATGTTAACACCCCGAGCGGCTCTTTGGTGTCCTCTGAGGCACAATTGTTTAATAAGCCA<br>TATTGGCTACAAAAAGCCCAGGGACATAACAATGGTATTTGTTGGGGTAATCAACTGTTT<br>GTTACTGTGGTAGATACCACACGCAGTACCAACATGACATTATGTGCATCCGTAACTACA<br>TCTTCCACATACACCAATTCTGATTATAAAGAGTACATGCGTCATGTGGAAGAGTATGAT<br>TTACAATTTATTTTTCAATTATGTAGCATTACATTGTCTGCTGAAGTAATGGCCTATATT<br>CACACAATGAATCCCTCTGTTTTGGAAGACTGGAACTTTGGGTTATCGCCTCCCCCAAAT<br>GGTACATTAGAAGATACCTATAGGTATGTGCAGTCACAGGCCATTACCTGTCAAAAGCCC<br>ACTCCTGAAAAGGAAAAGCCAGATCCCTATAAGAACCTTAGTTTTTGGGAGGTTAATTTA<br>AAAGAAAAGTTTTCTAGTGAATTGGATCAGTATCCTTTGGGACGCAAGTTTTTGTTACAA<br>AGTGGATATAGGGGACGGTCCTCTATTCGTACAGGTGTTAAGCGCCCTGCTGTTTCCAA
HPV11	Low-risk	CATCTACATACACCTAGTGGCTCATTGGTGTCTTCAGAGGCTCAAGCATCTAGTCAACCA<br>GATTGGCTTCAAAAGGCTCAGGGACATAACAATGGTATTTGCTGGGGAAACCACTTGTTT<br>GTTACTGTGGTAGATACCACACGCAGTACAAATATGACACTATGTGCATCTGTGTCTAAA<br>TCTGCTACATACACTAATTCAGATTATAAGGAATACATGCGCCATGTGGAGGAGTTTGAT<br>TTACAGTTTATTTTTCAATTGTGTAGCATTACATTATCTGCAGAAGTCATGGCCTATATA<br>CACACAATGAATCCTTCTGTTTTGGAGGACTGGAACTTTGGTTTATCGCCTCCACCAAAT<br>GGTACACTGGAGGATACTTATAGATATGTACAGTCACAGGCCATTACCTGTCAGAAACCC<br>ACACCTGAAAAAGAAAAACAGGATCCCTATAAGGATATGAGTTTTTGGGAGGTTAACTTA

		AAAGAAAAGTTTTCAAGTGAATTAGATCAGTTTCCCCTTGGACGTAAGTTTTTATTGCAAAGTGGATATCGAGGACGGACGTCTGCTCGTACAGGTATAAAGCGCCCAGCTCTCTGTAA
HPV16	High-risk	TATTTTCCTACACCTAGTGGTTCTATGGTTACCTCTGATGCCCAAATATTCAATAAACCTTATTGGTTACAACGAGCACAGGGCCACAATAATGGCATTTGTTGGGGTAACCAACTATTTGTTACTGTTGTTGATACTACACGCAGTACAAATATGTCATTATGTGCTGCCATATCTACTTCAGAAACTACATATAAAAATACTAACTTTAAGGAGTACCTACGACATGGGGAGGAATATGATTTACAGTTTATTTTTCAACTGTGCAAAATAACCTTAACTGCAGACGTTATGACATACATACATTCTATGAATTCCACTATTTTGGAGGACTGGAATTTTGGTCTACAACCTCCCCCAGGAGGCACACTAGAAGATACTTATAGGTTTGTAACCCAGGCAATTGCTTGTCAAAAACATACACCTCCAGCACCTAAAGAAGATGATCCCCTTAAAAAATACACTTTTTGGGAAGTAAATTTAAAGGAAAAGTTTTCTGCAGACCTAGATCAGTTTCCTTTAGGACGCAAATTTTTACTACAAGCAGGATTGAAGGCCAAACCAAAATTTACATTAGGAAAACGAAAAGCTACACCCACCAC
HPV18	High-risk	TATTCTCCCTCTCCAAGTGGCTCTATTGTTACCTCTGACTCCCAGTTGTTTAATAAACCATATTGGTTACATAAGGCACAGGGTCATAACAATGGTGTTTGCTGGCATAATCAATTATTTGTTACTGTGGTAGATACCACTCCCAGTACCAATTTAACAATATGTGCTTCTACACAGTCTCCTGTACCTGGGCAATATGATGCTACCAAATTTAAGCAGTATAGCAGACATGTTGAGGAATATGATTTGCAGTTTATTTTTCAGTTGTGTACTATTACTTTAACTGCAGATGTTATGTCCTATATTCATAGTATGAATAGCAGTATTTTAGAGGATTGGAACTTTGGTGTTCCCCCCCCCCCAACTACTAGTTTGGTGGATACATATCGTTTTGTACAATCTGTTGCTATTACCTGTCAAAAGGATGCTGCACCGGCTGAAAATAAGGATCCCTATGATAAGTTAAAGTTTTGGAATGTGGATTTAAAGGAAAAGTTTTCTTTAGACTTAGATCAATATCCCCTTGGACGTAAATTTTTGGTTCAGGCTGGATTGCGTCGCAAGCCCACCATAGGCCCTCGCAAACGTTCTGCTCCATCTGCCAC
HPV31	High-risk	TACTTTCCTACACCTAGCGGCTCCATGGTTACTTCAGATGCACAAATTTTTAATAAACCATATTGGATGCAACGTGCTCAGGGACACAATAATGGTATTTGTTGGGGCAATCAGTTATTTGTTACTGTGGTAGATACCACACGTAGTACCAATATGTCTGTTTGTGCTGCAATTGCAAAC

		AGTGATACTACATTTAAAAGTAGTAATTTTAAAGAGTATTTAAGACATGGTGAGGAATTT<br>GATTTACAATTTATATTTCAGTTATGCAAAATAACATTATCTGCAGACATAATGACATAT<br>ATTCACAGTATGAATCCTGCTATTTTGGAAGATTGGAATTTTGGATTGACCACACCTCCC<br>TCAGGTTCTTTGGAGGATACCTATAGGTTTGTCACCTCACAGGCCATTACATGTCAAAAA<br>ACTGCCCCCCAAAAGCCCAAGGAAGATCCATTTAAAGATTATGTATTTTGGGAGGTTAAT<br>TTAAAAGAAAAGTTTTCTGCAGATTTAGATCAGTTTCCACTGGGTCGCAAATTTTTATTA<br>CAGGCAGGATATAGGGCACGTCCTAAATTTAAAGCAGGTAAACGTAGTGCACCCTCAGCA<br>TC
HPV33	High-risk	TTTTTTCCCACTCCTAGTGGATCAATGGTTACTTCCGAATCTCAGTTATTTAATAAGCCA<br>TATTGGCTACAACGTGCACAAGGTCATAATAATGGTATTTGTTGGGGCAATCAGGTATTT<br>GTTACTGTGGTAGATACCACTCGCAGTACTAATATGACTTTATGCACACAAGTAACTAGT<br>GACAGTACATATAAAAATGAAAATTTTAAAGAATATATAAGACATGTTGAAGAATATGAT<br>CTACAGTTTGTTTTTCAACTATGCAAAGTTACCTTAACTGCAGAAGTTATGACATATATT<br>CATGCTATGAATCCAGATATTTTAGAAGATTGGCAATTTGGTTTAACACCTCCTCCATCT<br>GCTAGTTTACAGGATACCTATAGGTTTGTTACCTCTCAGGCTATTACGTGTCAAAAAACA<br>GTACCTCCAAAGGAAAAGGAAGACCCCTTAGGTAAATATACATTTTGGGAAGTGGATTTA<br>AAGGAAAAATTTTCAGCAGATTTAGATCAGTTTCCTTTGGGACGCAAGTTTTTATTACAG<br>GCAGGTCTTAAAGCAAAACCTAAACTTAAACGTGCAGCCCCCACATCCACCCGCACATC
HPV35	High-risk	TATTTTCCTACTCCTAGTGGCTCTATGGTAACCTCCGATGCACAAATATTTAATAAACCA<br>TATTGGTTGCAACGTGCACAAGGCCATAATAATGGTATTTGTTGGAGTAACCAATTGTTT<br>GTTACTGTAGTTGATACAACCCGTAGTACAAATATGTCTGTGTGTTCTGCTGTGTCTTCT<br>AGTGACAGTACATATAAAAATGACAATTTTAAGGAATATTTAAGGCATGGTGAAGAATAT<br>GATTTACAGTTTATTTTTCAGTTATGTAAAATAACACTAACAGCAGATGTTATGACATAT<br>ATTCATAGTATGAACCCGTCCATTTTAGAGGATTGGAATTTTGGCCTTACACCACCGCCT<br>TCTGGTACCTTAGAGGACACATATCGCTATGTAACATCACAGGCTGTAACTTGTCAAAAA<br>CCCAGTGCACCAAAACCTAAAGATGATCCATTAAAAAATTATACTTTTTGGGAGGTTGAT<br>TTAAAGGAAAAGTTTTCTGCAGACTTAGATCAATTTCCGTTGGGCCGTAAATTTTTGTTA

		CAAGCAGGACTAAAGGCCAGGCCTAATTTTAGATTAGGCAAGCGTGCAGCTCCAGCATCT
HPV39	High-risk	TACTGCCCCTCTCCCAGCGGTTCCATGGTAACCTCTGATTCCCAGTTATTTAATAAGCCT<br>TATTGGCTACATAAGGCCCAGGGCCACAACAATGGTATATGTTGGCATAATCAATTATTT<br>CTTACTGTTGTGGACACTACCCGTAGTACCAACTTTACATTATCTACCTCTATAGAGTCT<br>TCCATACCTTCTACATATGATCCTTCTAAGTTTAAGGAATATACCAGGCACGTGGAGGAG<br>TATGATTTACAATTTATATTTCAACTGTGTACTGTCACATTAACAACTGATGTTATGTCT<br>TATATTCACACTATGAATTCCTCTATATTGGACAATTGGAATTTTGCTGTAGCTCCTCCA<br>CCATCTGCCAGTTTGGTAGACACTTACAGATACCTACAGTCTGCAGCCATTACATGTCAA<br>AAGGATGCTCCAGCACCTGAAAAGAAAGATCCATATGACGGTCTAAAGTTTTGGAATGTT<br>GACTTAAGGGAAAAGTTTAGTTTGGAACTTGATCAATTCCCTTTGGGACGTAAATTTTTG<br>TTGCAGGCCAGGGTCCGCAGGCGCCCTACTATAGGTCCCCGAAAGCGGCCTGCTGCATCC<br>ACTTC
HPV42	Low-risk	TATTATCCTACCCCTAGTGGTTCTATGGTAACATCTGATGCACAACTATTTAATAAACCA<br>TATTGGTTACAACAAGCACAAGGACACAATAATGGTATATGTTGGGGAAATCAGCTATTT<br>TTAACTGTGGTTGATACTACCCGTAGTACTAACATGACTTTGTGTGCCACTGCAACATCT<br>GGTGATACATATACAGCTGCTAATTTTAAGGAATATTTAAGACATGCTGAAGAATATGAT<br>GTGCAATTTATATTTCAATTGTGTAAAATAACATTAACTGTTGAAGTTATGTCATATATA<br>CACAATATGAATCCTAACATATTAGAGGAGTGGAATGTTGGTGTTGCACCACCACCTTCA<br>GGAACTTTAGAAGATAGTTATAGGTATGTACAATCAGAAGCTATTCGCTGTCAGGCTAAG<br>GTAACAACGCCAGAAAAAAAGGATCCTTATTCAGACTTTTGGTTTTGGGAGGTAAATTTA<br>TCTGAAAAGTTTTCTACTGATTTAGATCAATTTCCTTTAGGTAGAAAGTTTTTACTGCAG<br>GCCGGGTTGCGTGCAAGGCCTAAACTGTCTGTAGGTAAACGAAAGGCGTCTACAGCTAA
HPV43	Low-risk	TATTTTTCTACACCCAGTGGGTCTTTGGTTACTTCTGATTCTCAATTGTTTAACAAACCC<br>TTATGGATACAAAAGGCCCAGGGACATAATAATGGCATTTGTTTTGGGAATCAGTTGTTT<br>GTTACAGTGGTAGATACCACTCGTAGTACAAACTTAACGTTATGTGCCTCTACTGACCCT<br>ACTGTGCCCAGTACATATGACAATGCAAAGTTTAAGGAATACCTGCGGCATGTGGAAGAA<br>TATGATCTGCAGTTTATATTTCAATTATGCATAATAACGCTAAACCCAGAGGTTATGACA

		TATATTCATACTATGGATCCCACATTATTAGAGGACTGGAATTTTGGTGTGTCCCCACCT<br>GCCTCTGCTTCTTTGGAAGATACTTATCGCTTTTTGTCTAACAAGGCCATTGCATGTCAA<br>AAAAATGCTCCCCCAAAAGAACGGGAGGATCCCTATAAAAAGTATACATTTTGGGATATA<br>AATCTTACAGAAAAGTTTTCTGCACAACTTACCCAGTTTCCCTTAGGGCGCAAATTTGTT<br>ATGCAGGCGGGTTTGCGTCCCAAACCTAAATTAAAAACTGTAAAGCGTTCTGCACCATCC<br>TCCTC
HPV44	Low-risk	TACTTTAATACACCCAGTGGTTCTCTTGTATCTTCTGAAACCCAATTATTTAATAAGCCT<br>TTTTGGTTGCAAAAGGCGCAGGGCCACAATAATGGTATTTGTTGGGGAAATCAGTTATTT<br>GTTACTGTTGTAGATACTACCCGTAGTACAAACATGACAATATGTGCTGCCACTACACAG<br>TCCCCTCCGTCTACATATACTAGTGAACAATATAAGCAATACATGCGACATGTTGAGGAG<br>TTTGACTTACAATTTATGTTTCAATTATGTAGTATTACCTTAACGGCGGAGGTAATGGCC<br>TATCTTCATACTATGAATGCTGGTATTTTAGAACAGTGGAACTTTGGGTTGTCGCCGCCC<br>CCAAATGGTACCTTAGAGGACAAATACAGATATGTGCAGTCCCAGGCCATTACATGTCAA<br>AAGCCACCCCCTGAAAAGGCAAAGCAGGACCCCTATGCAAAATTAAGTTTTTGGGAGGTG<br>GATCTTAGAGAAAAGTTTTCTAGTGAGTTGGATCAATATCCCCTTGGTAGAAAATTTTTA<br>TTACAAACGGGTGTGCAGGCCCGTTCCTCTGTTCGTGTGGGTAGGAAACGTCCTGCGTCT<br>GCAGC
HPV45	High-risk	TATTCCCCTTCTCCCAGTGGCTCTATTATTACTTCTGATTCTCAATTATTTAATAAGCCA<br>TATTGGTTACATAAGGCCCAGGGCCATAACAATGGTATTTGTTGGCATAATCAGTTGTTT<br>GTTACTGTAGTGGACACTACCCGCAGTACTAATTTAACATTATGTGCCTCTACACAAAAT<br>CCTGTGCCAAGTACATATGACCCTACTAAGTTTAAGCAGTATAGTAGACATGTGGAGGAA<br>TATGATTTACAGTTTATTTTTCAGTTGTGCACTATTACTTTAACTGCAGAGGTTATGTCA<br>TATATCCATAGTATGAATAGTAGTATATTAGAAAATTGGAATTTTGGTGTCCCTCCACCA<br>CCTACTACAAGTTTGGTGGATACATATCGTTTTGTGCAATCAGTTGCTGTTACCTGTCAA<br>AAGGATACTACACCTCCAGAAAAGCAGGATCCATATGATAAATTAAAGTTTTGGACTGTT<br>GACCTAAAGGAAAAATTTTCCTCCGATTTGGATCAATATCCCCTTGGTCGAAAGTTTTTA<br>GTTCAGGCTGGGTTACGTCGTAGGCCTACCATAGGACCTCGTAAGCGTCCTGCTGCTTCC

		ACGTC
HPV51	High-risk	TACTCTGCTACTCCCAGTGGGTCTATGATAACATCTGATTCTCAAATTTTTAATAAGCCT<br>TATTGGCTCCACCGTGCGCAGGGTCACAATAATGGCATTTGCTGGAACAATCAGCTTTTT<br>ATTACCTGTGTTGATACTACCAGAAGTACAAATTTAACTATTAGCACTGCCACTGCTGCG<br>GTTTCCCCAACATTTACTCCAAGTAACTTTAAGCAATATATTAGGCATGGGGAAGAGTAT<br>GAATTGCAATTTATTTTTCAATTATGTAAAATTACTTTAACTACAGAGGTAATGGCTTAT<br>TTACACACAATGGATCCTACCATTCTTGAACAGTGGAATTTTGGATTAACATTACCTCCG<br>TCTGCTAGTTTGGAGGATGCATATAGGTTTGTTAGAAATGCAGCTACTAGCTGTCAAAAG<br>GACACCCCTCCACAGGCTAAGCCAGATCCTTTGGCCAAATATAAATTTTGGGATGTTGAT<br>TTAAAGGAACGATTTTCTTTAGATTTAGACCAATTTGCATTGGGTCGCAAGTTTTTGTTG<br>CAGGTTGGCGTACAACGCAAGCCCAGACCAGGCCTTAAACGCCCGGCCTCATCGGCATCC<br>TCT
HPV52	High-risk	TTTTTTCCTACTCCTAGTGGTTCTATGGTAACCTCAGAATCCCAATTATTTAATAAACCG<br>TACTGGTTACAACGTGCGCAGGGCCACAATAATGGCATATGTTGGGGCAATCAGTTGTTT<br>GTCACAGTTGTGGATACCACTCGTAGCACTAACATGACTTTATGTGCTGAGGTTAAAAAG<br>GAAAGCACATATAAAAATGAAAATTTTAAGGAATACCTTCGTCATGGCGAGGAATTTGAT<br>TTACAATTTATTTTTCAATTGTGCAAAATTACATTAACAGCTGATGTTATGACATACATT<br>CATAAGATGGATGCCACTATTTTAGAGGACTGGCAATTTGGCCTTACCCCACCACCGTCT<br>GCATCTTTGGAGGACACATACAGATTTGTCACTTCTACTGCTATAACTTGTCAAAAAAAC<br>ACACCACCTAAAGGAAAGGAAGATCCTTTAAAGGACTATATGTTTTGGGAGGTGGATTTA<br>AAAGAAAAGTTTTCTGCAGATTTAGATCAGTTTCCTTTAGGTAGGAAGTTTTTGTTACAG<br>GCAGGGCTACAGGCTAGGCCCAAACTAAAACGCCCTGCATCATCGGCCCCACGTACCTC
HPV56	High-risk	ATGTTGCTACGCCTAGTGGGTCTATGATTACGTCTGAGGCACAGTTATTTAATAAACCTT<br>ATTGGTTGCAACGTGCCCAAGGCCATAATAATGGCATTTGCTGGGGTAATCAATTATTTG<br>TTACTGTAGTAGATACTACTAGAAGTACTAACATGACTATTAGTACTGCTACAGAACAGT<br>TAAGTAAATATGATGCACGAAAAATTAATCAGTACCTTAGACATGTGGAGGAATATGAAT<br>TACAATTTGTTTTTCAATTATGCAAAATTACTTTGTCTGCAGAGGTTATGGCATATTTAC

		ATAATATGAATGCTAACCTACTGGAGGACTGGAATATTGGGTTATCCCCGCCAGTGGCCA<br>CCAGCCTAGAAGATAAATATAGATATGTTAGAAGCACAGCTATAACATGTCAACGGGAAC<br>AGCCACCAACAGAAAAACAGGACCCATTAGCTAAATATAAATTTTGGGATGTTAACTTAC<br>AGGACAGTTTTTCTACAGACCTGGATCAATTTCCACTGGGTAGAAAATTTTTAATGCAAC<br>TGGGCACTAGGTCAAAGCCTGCTGTAGCTACCTCTAAAAAGCGATCTGCTCCTACCTC
HPV58	High-risk	TTTTTTCCAACTCCTAGTGGCTCTATAGTTACCTCAGAATCACAATTATTTAATAAGCCT<br>TATTGGCTACAGCGTGCACAAGGTCATAACAATGGCATTTGCTGGGGCAATCAGTTATTT<br>GTTACCGTGGTTGATACCACTCGTAGCACTAATATGACATTATGCACTGAAGTAACTAAG<br>GAAGGTACATATAAAAATGATAATTTTAAGGAATATGTACGTCATGTTGAAGAATATGAC<br>TTACAGTTTGTTTTTCAGCTTTGCAAAATTACACTAACTGCAGAGATAATGACATATATA<br>CATACTATGGATTCCAATATTTTGGAGGACTGGCAATTTGGTTTAACACCTCCTCCGTCT<br>GCCAGTTTACAGGACACATATAGATTTGTTACCTCCCAGGCTATTACTTGCCAAAAAACA<br>GCACCCCCTAAAGAAAAGGAAGATCCATTAAATAAATATACTTTTTGGGAGGTTAACTTA<br>AAGGAAAAGTTTTCTGCAGATCTAGATCAGTTTCCTTTGGGACGAAAGTTTTTATTACAA<br>TCAGGCCTTAAAGCAAAGCCCAGACTAAAACGTTCGGCCCCTACTACCCGTGCACCATC
HPV59	High-risk	TATTCCCCTTCCCCAAGTGGGTCTGTGGTTACTTCTGATTCACAATTATTTAATAAACCA<br>TATTGGCTGCACAAGGCTCAGGGTTTAAACAATGGTATATGTTGGCACAATCAATTGTTT<br>TTAACAGTTGTAGATACTACTCGCAGCACCAATCTTTCTGTGTGTGCTTCTACTACTTCT<br>TCTATTCCTAATGTATACACACCTACCAGTTTTAAAGAATATGCCAGACATGTGGAGGAA<br>TTTGATTTGCAGTTTATATTTCAACTGTGTAAAATAACATTAACTACAGAGGTAATGTCA<br>TACATTCATAATATGAATACCACTATTTTGGAGGATTGGAATTTTGGTGTTACACCACCT<br>CCTACTGCTAGTTTAGTTGACACATACCGTTTTGTTCAATCTGCTGCTGTAACTTGTCAA<br>AAGGACACCGCACCGCCAGTTAAACAGGACCCTTATGACAAACTAAAGTTTTGGCCTGTA<br>GATCTTAAGGAAAGGTTTTCTGCAGATCTTGATCAGTTTCCTTTGGGACGTAAATTTTTA<br>TTGCAATTAGGAGCTAGACCTAAGCCCACTATAGGCCCACGCAAACGTGCAGCGCCTGCC<br>CCTAC
HPV66	High-risk	TATGTTGCTACTCCTAGTGGGTCCATGATTACCTCTGAGGCCCAATTATTTAATAAACCT

		TATTGGTTGCAACGTGCACAGGGCCATAATAATGGCATATGCTGGGGTAATCAGGTATTT<br>GTTACTGTTGTGGATACTACCAGAAGCACCAACATGACTATTAATGCAGCTAAAAGCACA<br>TTAACTAAATATGATGCCCGTGAAATCAATCAATACCTTCGCCATGTGGAGGAATATGAA<br>CTACAGTTTGTGTTTCAACTTTGTAAAATAACCTTAACTGCAGAAGTTATGGCATATTTG<br>CATAATATGAATAATACTTTATTAGACGATTGGAATATTGGCTTATCCCCACCAGTTGCA<br>ACTAGCTTAGAGGATAAATATAGGTATATTAAAAGCACAGCTATTACATGTCAGAGGGAA<br>CAGCCCCCTGCAGAAAAGCAGGATCCCCTGGCTAAATATAAGTTTTGGGAAGTTAATTTA<br>CAGGACAGCTTTTCTGCAGACCTGGATCAGTTTCCTTTGGGTAGAAAATTTTTAATGCAA<br>CTAGGCCCTAGACCCCCTAGACCCAAGGCTAGTGTATCTGCCTCTAAAAGGCGGGCGGC
HPV68a	High-risk	TATGCCCCCTCGCCTAGCGGGTCTATGGTGTCCTCTGACTCCCAGTTATTTAACAAGCCC<br>TATTGGCTGCACAAGGCACAGGGACACAACAATGGTATTTGTTGGCATAATCAATTATTT<br>CTTACCGTTGTGGATACAACGCGCAGTACTAATTTTACATTGTCCACTACTACAGACTCT<br>ACTGTACCAGCTGTGTATGATTCTAATAAATTTAAGGAATATGTTAGGCATGTTGAGGAA<br>TATGATTTGCAGTTTATATTTCAGTTGTGTACTATAACATTATCCACTGATGTAATGTCA<br>TATATACATACTATGAATCCTGCTATTTTGGATGATTGGAATTTTGGTGTTGCCCCTCCA<br>CCATCTGCTAGTCTTGTAGATACATACCGCTACCTACAATCAGCAGCAATTACATGTCAA<br>AAGGACGCCCCTGCACCTGTTAAAAAAGATCCCTATGATGGTCTTAACTTTTGGAATGTG<br>GATTTAAAGGAAAAGTTTAGTTCTGAACTGGACCAATTCCCATTAGGACGCAAATTTCTG<br>TTACAGGCAGGTGTTCGCAGACGGCCCACCATAGGCCCTCGTAAACGCACTGCCACTGCA<br>GCTAC
HPV68b	High-risk	TATGCCCCCTCGCCTAGTGGGTCTATGGTATCCTCAGACTCCCAGTTATTTAACAAGCCC<br>TATTGGCTGCACAAGGCACAGGGACACAACAATGGTATTTGTTGGCATAATCAATTATTT<br>CTTACTGTTGTGGATACCACTCGCAGTACCAATTTTACTTTGTCTACTACTACTGAATCA<br>GCTGTACCAAATATTTATGATCCTAATAAATTTAAGGAATATATTAGGCATGTTGAGGAA<br>TATGATTTGCAATTTATATTTCAGTTGTGTACTATAACATTGTCCACTGATGTAATGTCC<br>TATATACATACTATGAATCCTGCTATTTTGGATGATTGGAATTTTGGTGTTGCCCCTCCA<br>CCATCTGCTAGTCTTGTAGATACATACCGCTATCTGCAATCAGCAGCAATTACATGTCAA

		AAAGACGCCCCTGCACCTACTAAAAAGGATCCATATGATGGCTTAAACTTTTGGAATGTA<br>AATTTAAAGGAAAAGTTTAGTTCTGAACTGGACCAGTTTCCTTTAGGACGCAAATTTCTT<br>TTACAGGCAGGCGTCCGCCGACGACCCACTATAGGCCCCCGTAAACGCCCCGCCACAGCA<br>ACTAC
