# Supplementary material for: A novel sequencing-based vaginal health assay combining self-sampling, HPV detection and genotyping, STI detection, and vaginal microbiome analysis
Source: PLoS One. 2019 May 1;14(5):e0215945. doi: 10.1371/journal.pone.0215945 (PMC6493738; doi:10.1371/journal.pone.0215945)
Supplement: S6 Table — The table shows the lowest dilution at which at least two of the three replicates had 2 or more reads per taxon, and the calculated threshold for identification per taxa at the LOD in number of reads. (PDF) [file pone.0215945.s008.pdf]

Supplementary material belonging to

"A novel sequencing-based vaginal health assay combining self-sampling, HPV detection and genotyping, STI detection, and vaginal microbiome analysis"

S6 Table. Limit of detection (LOD) assay for the bacterial targets. The table shows the lowest dilution at which at least two of the three replicates had 2 or more reads per taxon, and the calculated threshold for identification per taxa at the LOD in number of reads.

Taxon	Dilution	Rep1	Rep2	Rep3	Threshold
Aerococcus	1:500	6	3	5	50.3
Aerococcus christensenii	1:500	3	6	0	52.3
Atopobium	1:500	8	4	6	51.0
Atopobium vaginae	1:250	8	8	7	49.0
Chlamydia trachomatis	1:250	2	2	0	49.8
Dialister micraerophilus	1:250	4	5	5	49.0
Gardnerella	1:500	5	3	0	51.7
Gardnerella vaginalis	1:250	0	9	4	54.3
Gemella	1:250	5	10	9	51.8
Lactobacillus iners	1:250	15	4	7	55.9
Lactobacillus jensenii	1:500	7	8	7	49.0
Megasphaera	1:500	17	20	13	53.0
Mobiluncus curtisii	1:1000	15	17	19	51.0
Mycoplasma genitalium	1:100	2	8	3	52.6
Neisseria gonorrhoeae	1:250	0	11	5	55.7

Papillibacter	1:500	10	5	5	52.2
Parvimonas	1:500	4	3	0	51.1
Peptoniphilus	1:250	7	2	3	51.8
Peptostreptococcus	1:1000	3	5	7	51.0
Porphyromonas	1:100	16	0	9	59.1
Prevotella	1:1000	2	5	5	50.6
Prevotella amnii	1:500	14	10	19	54.3
Prevotella timonensis	1:1000	5	6	0	52.6
Sneathia	1:500	11	7	9	51.0
Staphylococcus aureus	1:500	4	10	7	52.3
Streptococcus agalactiae	1:500	7	4	4	50.6
