# Supplementary material for: A novel sequencing-based vaginal health assay combining self-sampling, HPV detection and genotyping, STI detection, and vaginal microbiome analysis
Source: PLoS One. 2019 May 1;14(5):e0215945. doi: 10.1371/journal.pone.0215945 (PMC6493738; doi:10.1371/journal.pone.0215945)
Supplement: S7 Table — The table shows the lowest dilution at which at least two of the three replicates had 2 or more reads per HPV type, and the calculated threshold for identification per HPV type at the LOD in number of reads. (PDF) [file pone.0215945.s009.pdf]

Supplementary material belonging to

"A novel sequencing-based vaginal health assay combining self-sampling, HPV detection and genotyping, STI detection, and vaginal microbiome analysis"

S7 Table. Limit of detection (LOD) assay for the HPV targets. The table shows the lowest dilution at which at least two of the three replicates had 2 or more reads per HPV type, and the calculated threshold for identification per HPV type at the LOD in number of reads.

HPV type	Dilution	Rep1	Rep2	Rep3	Threshold
6	1:100	27	75	6	87.7
11	1:100	41	61	8	76.1
16	1:100	7	0	9	46.4
18	1:100000	1	8	0	47.7
31	1:1	240	0	5	224.8
33	1:100	0	13	18	52.5
39	1:100000	0	24	10	56.2
42	1:100	27	16	0	58.3
44	1:100000	35	11	0	64.1
45	1:100	46	18	8	66.5
51	1:100000	0	2	2	41.6
52	1:100	100	8	0	114.9
56	1:100	3	2	2	40.8
58	1:100	22	13	7	50.2
59	1:100	0	14	23	55.6

66	1:100000	2	0	2	41.5
68a	1:1	307	145	219	149.3
68b	1:1	47	38	0	73.6
